# Supplementary material for: Meta-analysis of the impacts of global change factors on soil microbial diversity and functionality
Source: Nat Commun. 2020 Jun 17;11:3072. doi: 10.1038/s41467-020-16881-7 (PMC7300008; doi:10.1038/s41467-020-16881-7)
Supplement: Supplementary file 1 — Supplementary Information [file 41467_2020_16881_MOESM1_ESM.pdf]

Supplementary Information

Meta-analysis of the impacts of global change factors on soil microbial diversity and functionality

Zhou et al.

Includes:

Supplementary Tables (1-2)

Supplementary Figures (1-13)

Supplementary References (1)

Supplementary Table 1 Summary of dominant drivers of soil microbial alpha diversity in terrestrial ecosystems from large-scale studies.

Driver	Effect	Involved mechanism or theory	Location (sample size)
Temperature	Positive	Metabolic theory: Elevated temperature increases biodiversity by accelerating the biochemical reactions that control speciation rate[1].	North America (126 soils)[2]
Soil resource content	Positive	Species energy theory: Species richness increases monotonically with the energy or resource[3]; It assumes that diversity mirrors productivity within microorganisms because soil microbial biomass much depends upon the soil carbon or nutrient contents[4,5].	Global (~600 soils)[4]
Soil carbon to nutrient ratios	Negative	Stoichiometry theory: Fast growing microorganisms require higher demand for nutrients than plants[6]; It assumes that microbial diversity mirrors its biomass, and thus lower carbon to nutrient ratios may result in higher microbial diversity[4].	Scotland (179 sites)[7]
Plant diversity	Positive	Aboveground–belowground interactions: Plant diversity promotes the diversity of soil microbes by increasing the diversity of soil exudates and litter, physical microhabitats and environmental conditions, and plant hosts for symbiotic and pathogenic microbes[8,9,10].	Four continents (25 temperate grassland sites)[9]
Soil pH	Unimodal	Niche imposes a physiological constraint, altering competitive outcomes or reducing net growth of individual taxa unable to survive if the soil pH falls outside a certain range[11,12,13].	North and South America (98 soils[11]; 88 soils[12]); Arctic (29 sites)[13]
Aridity	Negative	Aridity decreases microbial diversity by its negative impact on soil carbon contents.	Global (80 dryland sites)[14]
Latitude	Negative	Covariant factors with latitudinal gradients, especially temperature and precipitation.	Global (365 soils)[15]; Southern hemisphere (647 sites)[16]

References:

[1]Allen, A. P., Brown, J. H. & Gillooly, J. F. Global biodiversity, biochemical kinetics, and the energetic-equivalence rule. *Science* **297**, 1545–1548 (2002).

[2]Zhou, J. Z. et al. Temperature mediates continental-scale diversity of microbes in forest soils. *Nat. Commun.* **7**, 12083 (2016).

[3]Cardinale, B. J., Hillebrand, H., Harpole, W., Gross, K. & Ptacnik, R. Separating the influence of resource 'availability' from resource 'imbalance' on productivity–diversity relationships. *Ecol. Lett.* **12**, 475–487 (2009).

[4]Delgado-Baquerizo, M. et al. Carbon content and climate variability drive global soil bacterial diversity patterns. *Ecol. Monogr.* **86**, 373–390 (2016).

[5]Xu, X., Thornton, P. E., & Post, W. M. A global analysis of soil microbial biomass carbon, nitrogen and phosphorus in terrestrial ecosystems. *Glob. Ecol. Biogeogr.* **22**, 737–749 (2013).

[6]Zechmeister-Boltenstern, S. et al. The application of ecological stoichiometry to plant–microbial–soil organic matter transformations. *Ecol. Monogr.* **85**, 133–155 (2015).

[7]Delgado-Baquerizo, M. et al. It is elemental: soil nutrient stoichiometry drives bacterial diversity. *Environ. Microbiol.* **19**, 1176–1188 (2017).

[8]Hooper, D. et al. Interactions between aboveground and belowground biodiversity in terrestrial ecosystems: patterns, mechanisms, and feedbacks. *Bioscience* **50**, 1049–1061 (2000).

[9]Prober, S. M. et al. Plant diversity predicts beta but not alpha diversity of soil microbes across grasslands worldwide. *Ecol. Lett.* **18**, 85–95 (2015).

[10]Eisenhauer, N. et al. Plant diversity effects on soil microorganisms support the singular hypothesis. *Ecology* **91**, 485–496 (2010).

[11]Fierer, N. & Jackson, R. B. The diversity and biogeography of soil bacterial communities. *Proc. Natl Acad. Sci. USA* **103**, 626–631 (2006).

[12]Lauber, C. L., Hamady, M., Knight, R. & Fierer, N. Pyrosequencing-based assessment of soil pH as a predictor of soil bacterial community structure at the continental scale. *Appl. Environ. Microbiol.* **75**, 5111–5120 (2009).

[13]Chu, H. Y. et al. Soil bacterial diversity in the Arctic is not fundamentally different from that found in other biomes. *Environ. Microbiol.* **12**, 2998–3006, (2010).

[14]Maestre, F. T. et al. Increasing aridity reduces soil microbial diversity and abundance in global drylands. *Proc. Natl Acad. Sci. USA* **112**, 15684–15689 (2015).

[15]Tedersoo, L. et al. Global diversity and geography of soil fungi. *Science* **346**, 1256688 (2014).

[16]Delgado-Baquerizo, M. et al. Ecological drivers of soil microbial diversity and soil biological networks in the Southern Hemisphere. *Ecology* **99**, 583–596 (2018).

Supplementary Table 2 Correlation coefficients between response of microbial alpha diversity and climate factors.

GCFs	Climates	RR of richness	RR of Shannon index
W	MAT	-0.01(*n* = 100)	0.07(*n* = 73)
	MAP	-0.11(*n* = 100)	-0.05(*n* = 73)
eCO_2	MAT	-0.18(*n* = 39)	0.31(*n* = 22)
	MAP	-0.31(*n* = 39)	0.09(*n* = 22)
PPT-	MAT	-0.16(*n* = 47)	0.15(*n* = 59)
	MAP	0.00(*n* = 47)	0.03(*n* = 59)
PPT+	MAT	-0.25(*n* = 47)	-0.09(*n* = 44)
	MAP	-0.29(*n* = 47)	-0.1(*n* = 44)
P	MAT	-0.12(*n* = 62)	-0.29(*n* = 35)
	MAP	-0.07(*n* = 62)	0.13(*n* = 35)
N	MAT	**-0.14(*n* = 287)***	**-0.2(*n* = 199)****
	MAP	-0.07(*n* = 287)	0.07(*n* = 199)
LUC	MAT	-0.05(*n* = 291)	0.09(*n* = 246)
	MAP	0.00(*n* = 292)	0.07(*n* = 246)
W×eCO_2	MAT	-0.04(*n* = 9)	-0.38(*n* = 5)
	MAP	0.13(*n* = 9)	0.48(*n* = 5)
N×PPT+	MAT	**-0.51(*n* = 25)****	**-0.64(*n* = 23)*****
	MAP	**-0.71(*n* = 25)*****	**-0.71(*n* = 23)*****
N×P	MAT	**-0.28(*n* = 58)***	-0.16(*n* = 37)
	MAP	**-0.35(*n* = 58)****	-0.14(*n* = 37)
N×P×K	MAT	**-0.21(*n* = 101)***	0.05(*n* = 93)
	MAP	-0.08(*n* = 101)	0.07(*n* = 93)

GCFs, global change factors (including warming (W), carbon dioxide enrichment (eCO_2), decreased precipitation (PPT-), increased precipitation (PPT+), phosphorous addition (P), nitrogen addition (N), land use change (LUC), W×eCO_2, N×PPT+, N plus P plus potassium addition (N×P×K)); MAT, mean annual temperature (℃); MAP, mean annual precipitation (mm). The significant correlations are marked by asterisk (*, P<0.05; **, P<0.01; ***, P<0.001).

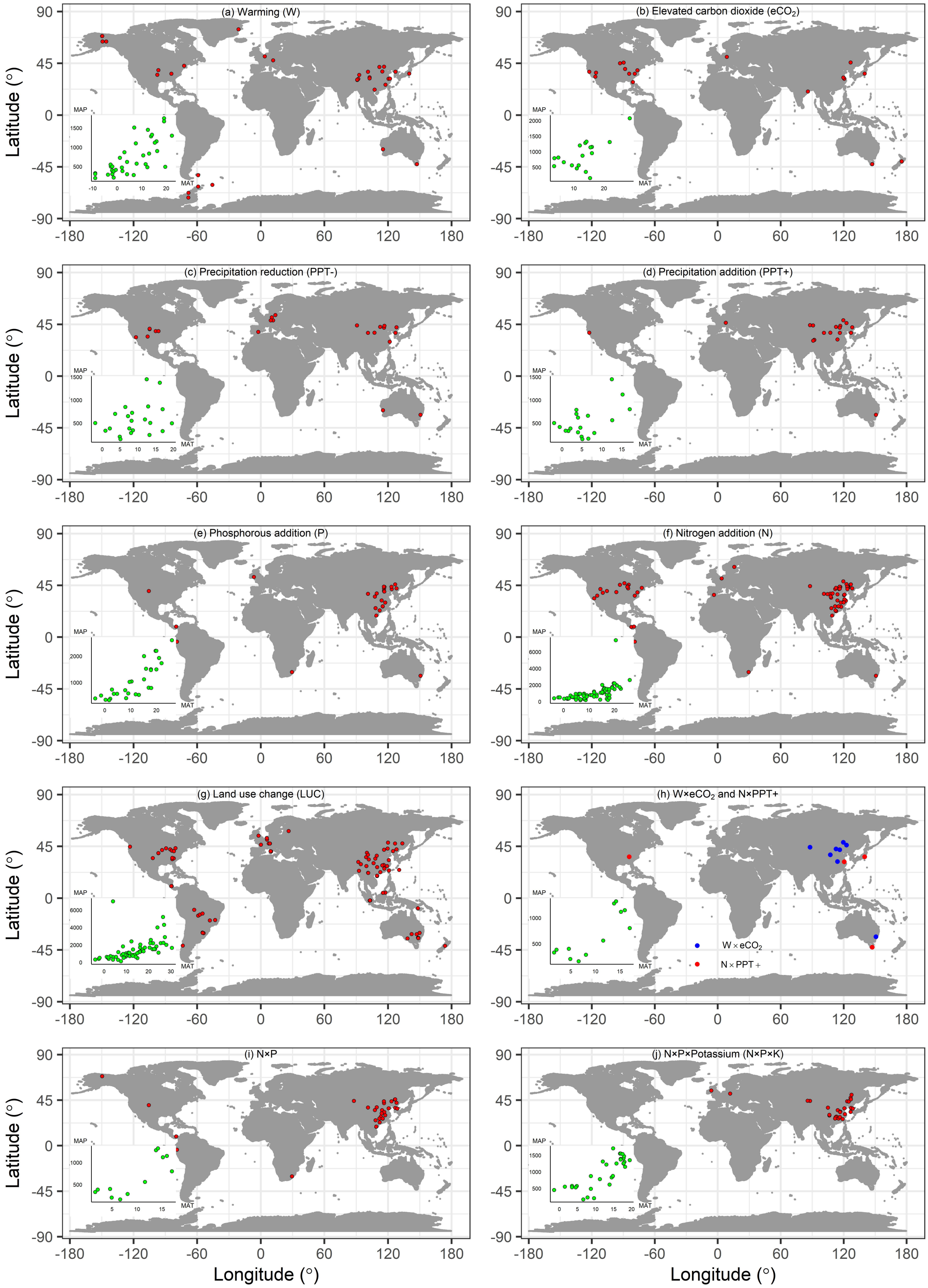

Supplementary Fig. 1 Distribution of the sampling sites for different global change factors. The inset scatter plots show the relationships between mean annual temperature (MAT, ℃) and mean annual precipitation (MAP, mm). Source data are provided as a Source Data file.

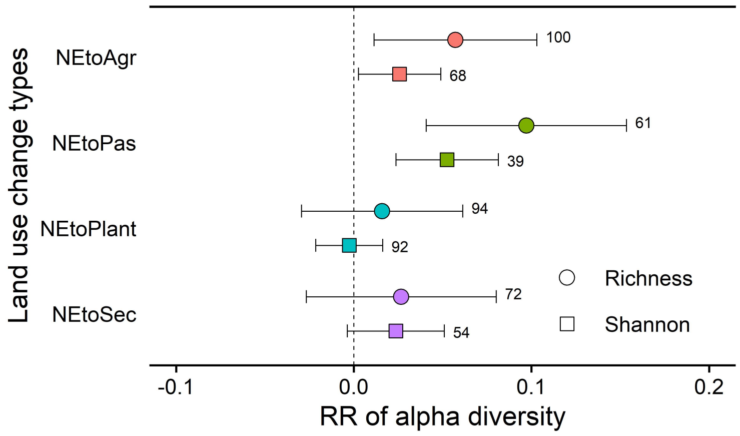

Supplementary Fig. 2 Responses of microbial alpha diversity to different land use change types. Weighted means and their 95% confidence intervals of response ratios (RR) are given. The numbers at the right side of the confidence intervals represent the sample sizes. NEtoAgr, conversion from native ecosystem to agriculture, NEtoPas, conversion from native ecosystem to pasture, NEtoPlant, conversion from native ecosystem to plantation, NEtoSec, conversion from native ecosystem to secondary ecosystem. Source data are provided as a Source Data file.

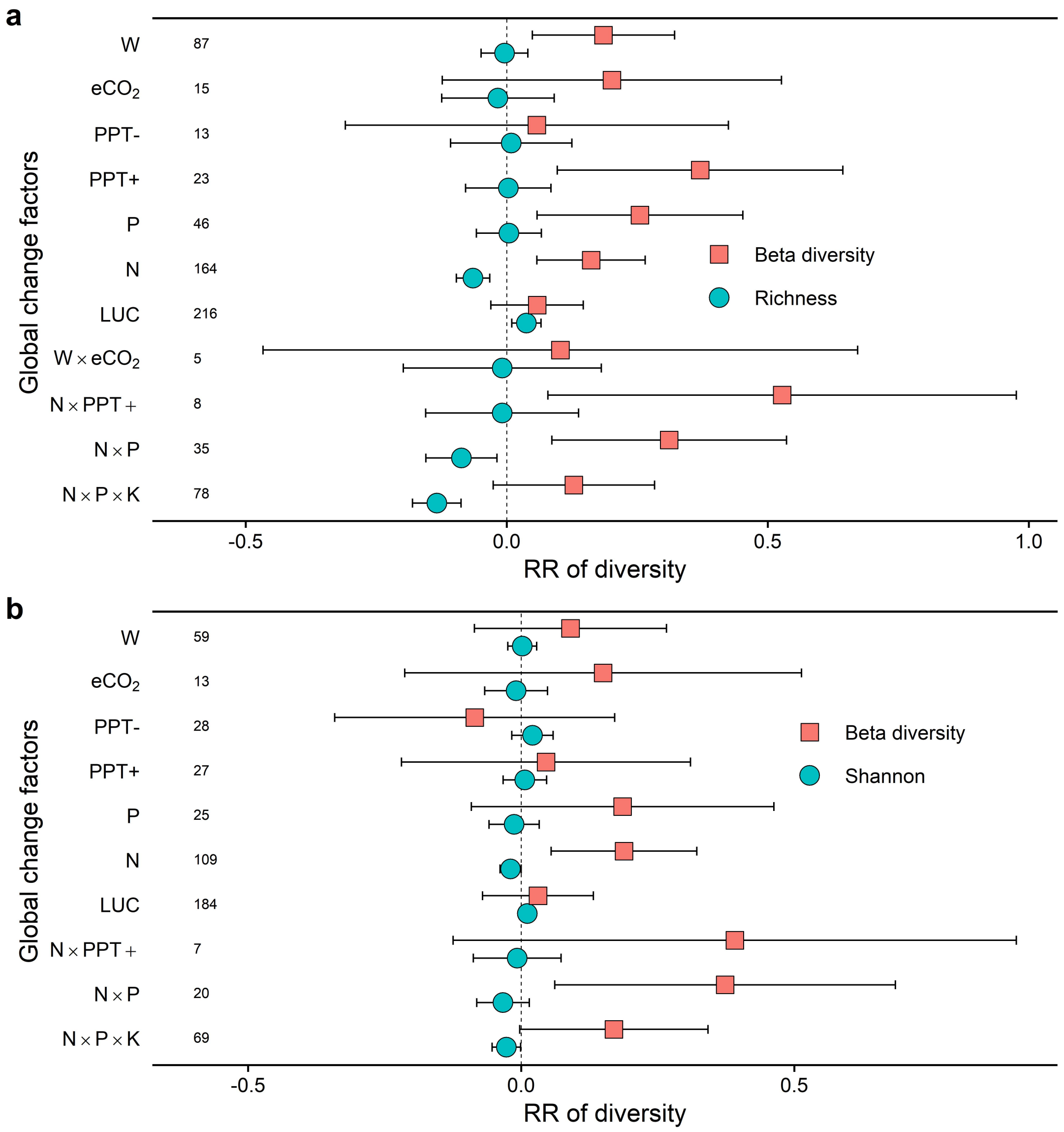

Supplementary Fig. 3 Paired comparisons of the responses of microbial alpha diversity and beta diversity to global change factors. **a** Comparisons between response ratio (RR) of richness and RR of beta diversity. **b** Comparisons between RR of Shannon index and RR of beta diversity. Weighted means and their 95% confidence intervals of RRs are given. The numbers at the left represent the sample sizes. W, warming; eCO_2, carbon dioxide enrichment; PPT-, decreased precipitation; PPT+, increased precipitation; P, phosphorous addition; N, nitrogen addition; LUC, land use change; W×eCO_2, warming plus carbon dioxide enrichment; N×PPT+, nitrogen addition plus increased precipitation; N×P, nitrogen plus phosphorous addition; N×P×K, nitrogen plus phosphorous plus potassium addition. Source data are provided as a Source Data file.

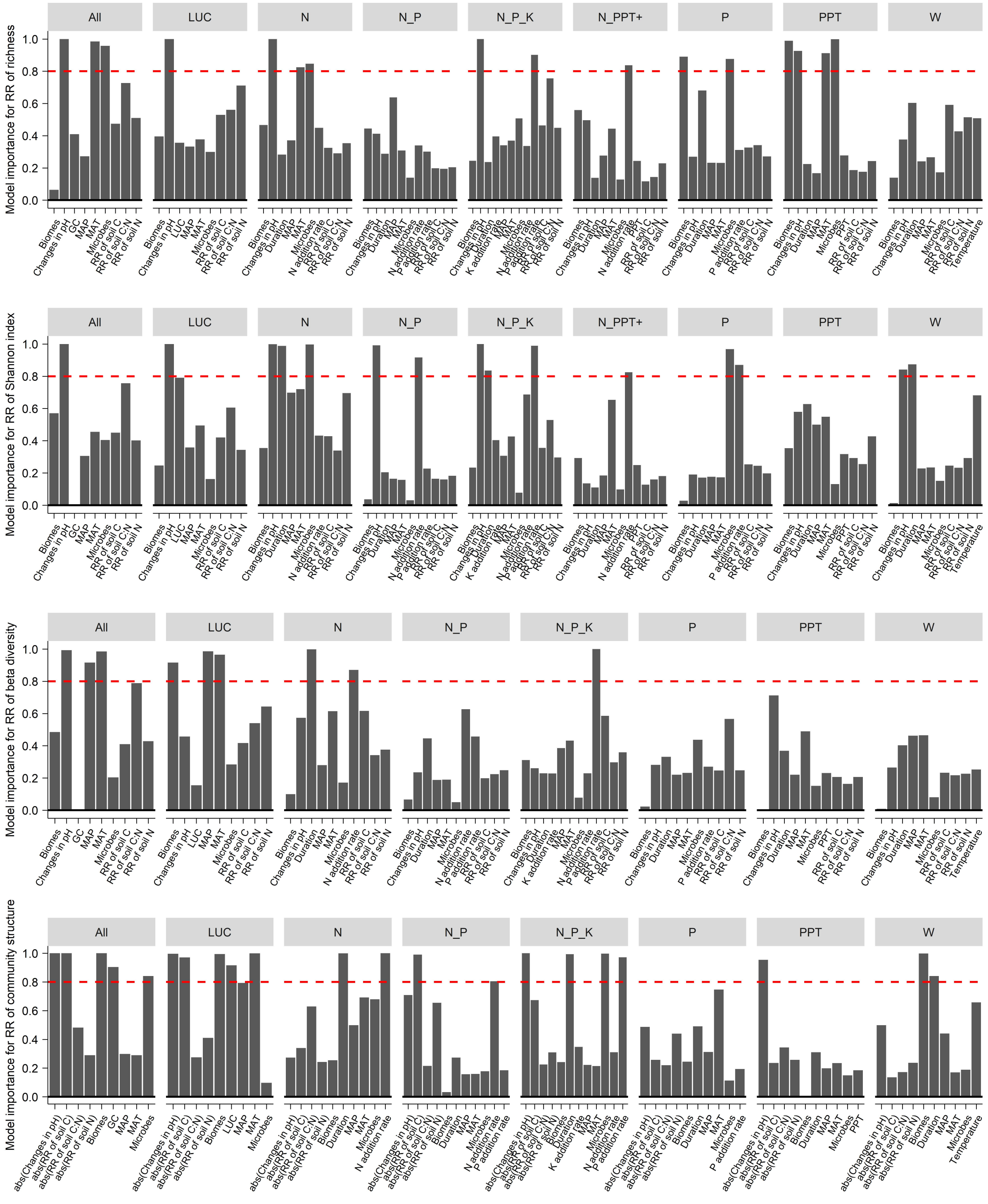

Supplementary Fig. 4 Model-averaged importance of the predictors of global changes effect on microbial communities. The importance is based on the sum of Akaike weights derived from the model selection using AIC (Akaike's Information Criteria corrected for small samples). A cutoff of 0.8 (the red dashed line) is set to differentiate between important and non-essential predictors. abs, absolute value. LUC, land use change; N, nitrogen addition; N_P, nitrogen plus phosphorous addition; N_P_K, nitrogen plus phosphorous plus potassium addition; N×PPT+, nitrogen addition plus increased precipitation; P, phosphorous addition; PPT, altered precipitation; W, warming. Source data are provided as a Source Data file.

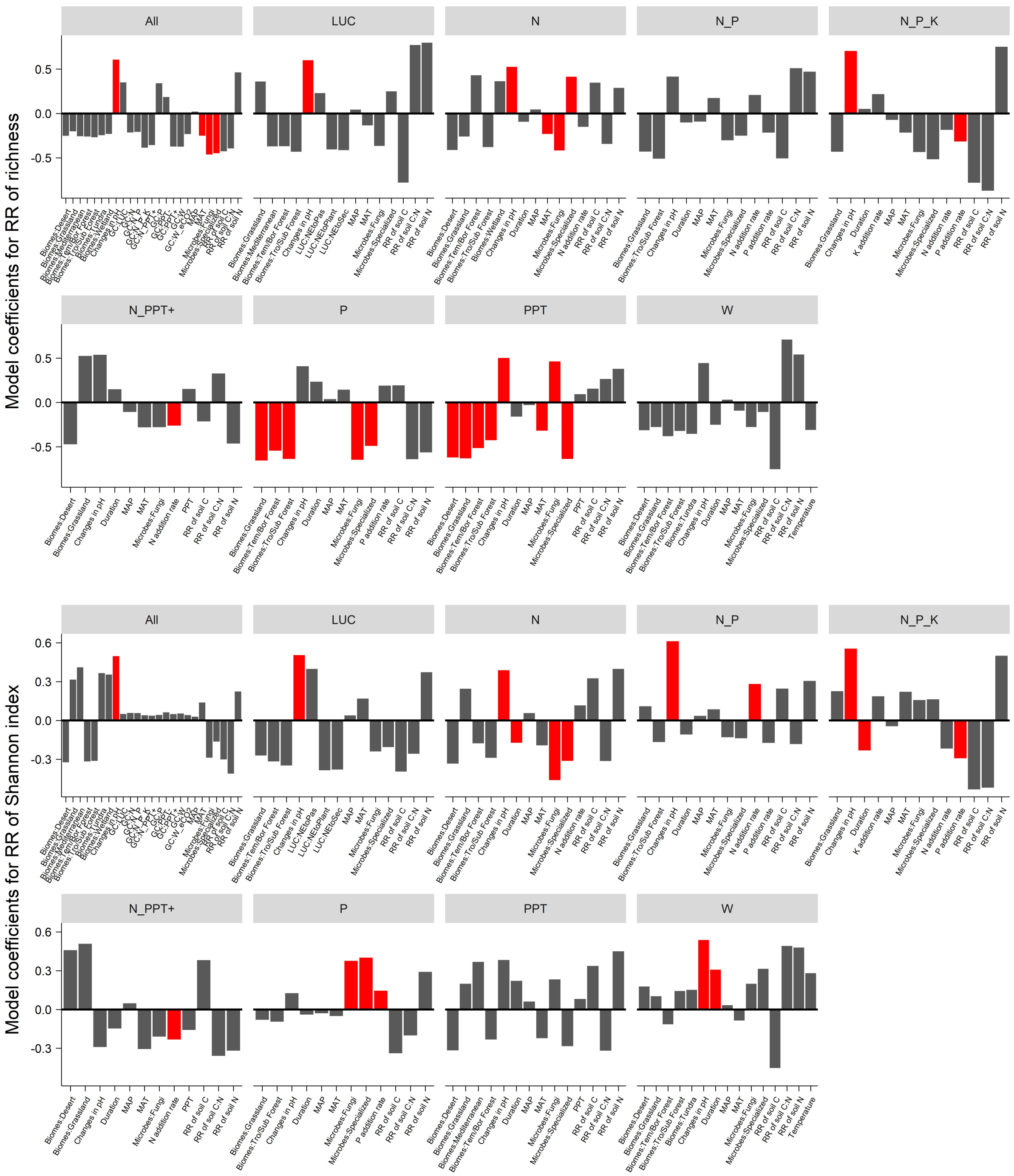

Supplementary Fig. 5 Weighted averages of the model coefficients across various models for the responses of microbial alpha diversity to global change factors. The weights equal to the model probabilities. Model parameters are transferred by the fourth root for better visualization and ordered by increasing relative importance. The predictors in red bars are the ones with the sum of Akaike weights > 0.8. LUC, land use change; N, nitrogen addition; N_P, nitrogen plus phosphorous addition; N_P_K, nitrogen plus phosphorous plus potassium addition; N×PPT+, nitrogen addition plus increased precipitation; P, phosphorous addition; PPT, altered precipitation; W, warming. MAT, mean annual temperature (℃); MAP, mean annual precipitation (mm). The units for predictors of duration, N/P/K addition rates, altered PPT, and elevated temperature are year, g N/P/K m^{-2} $year^{-1}$, %, and ℃, respectively. Source data are provided as a Source Data file.

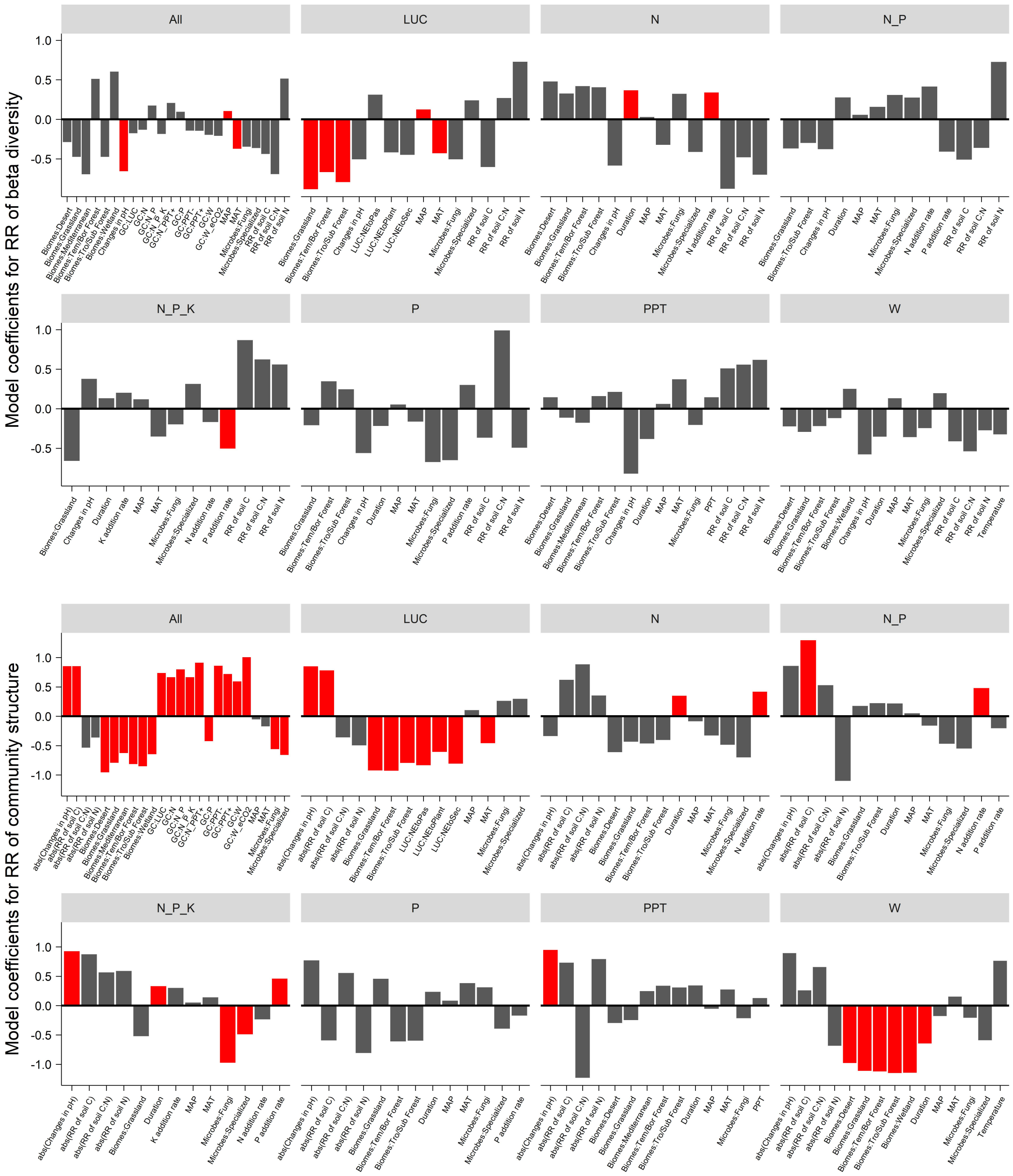

Supplementary Fig. 6 Weighted averages of the model coefficients across various models for the responses of microbial beta diversity and community structure to global change factors. The weights equal to the model probabilities. Model parameters are transferred by the fourth root for better visualization and ordered by increasing relative importance. The predictors in red bars are the ones with the sum of Akaike weights > 0.8. LUC, land use change; N, nitrogen addition; N_P, nitrogen plus phosphorous addition; N_P_K, nitrogen plus phosphorous plus potassium addition; N×PPT+, nitrogen addition plus increased precipitation; P, phosphorous addition; PPT, altered precipitation; W, warming. MAT, mean annual temperature (°C); MAP, mean annual precipitation (mm). The units for predictors of duration, N/P/K addition rates, altered PPT, and elevated temperature are year, g N/P/K m^{-2} $year^{-1}$, %, and °C, respectively. Source data are provided as a Source Data file.

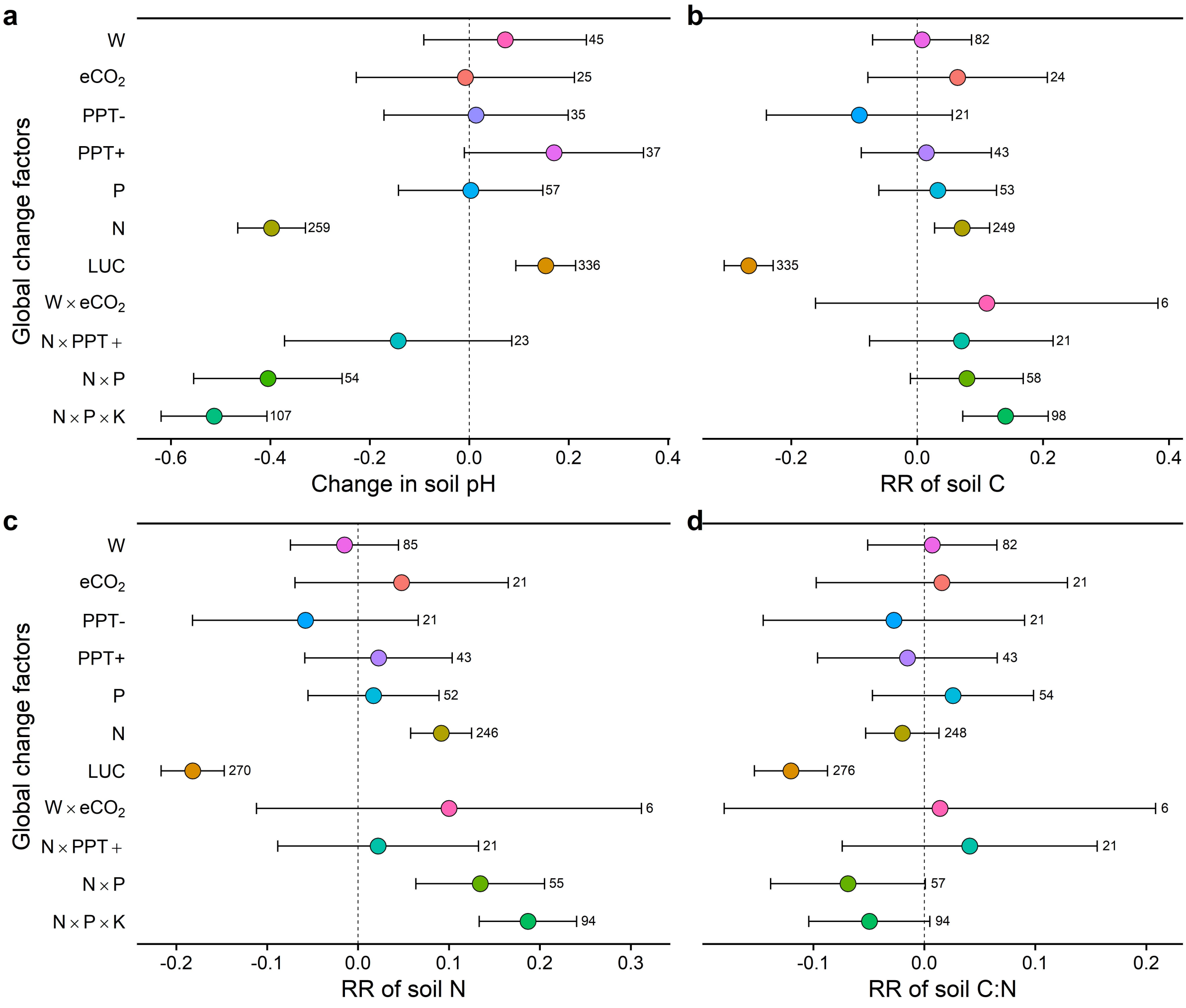

e

r values	RR of soil C	RR of soil N	RR of soil C:N
Change in soil pH	-0.37***(*n* = 910)	-0.20***(*n* = 831)	-0.18***(*n* = 843)
RR of soil C		0.72***(*n* = 911)	0.42***(*n* = 911)
RR of soil N			-0.33***(*n* = 911)

Supplementary Fig. 7 Global change factors (GCFs) induced changes in soil properties and their correlations. **a** GCFs induced change in soil pH. **b** Response ratio (RR) of soil C to GCFs. **c** RR of soil N to GCFs. **d** RR of soil C:N to GCFs. Weighted means and their 95% confidence intervals of RRs are given. The numbers at the right side of the confidence intervals represent the sample sizes. W, warming; eCO_2, carbon dioxide enrichment; PPT-, decreased precipitation; PPT+, increased precipitation; P, phosphorous addition; N, nitrogen addition; LUC, land use change; W×eCO_2, warming plus carbon dioxide enrichment; N×PPT+, nitrogen addition plus increased precipitation; N×P, nitrogen plus phosphorous addition; N×P×K, nitrogen plus phosphorous plus potassium addition. **e** Pearson's *r* correlation coefficients among GCFs induced shifts in soil properties. ***, P<0.001. Source data are provided as a Source Data file.

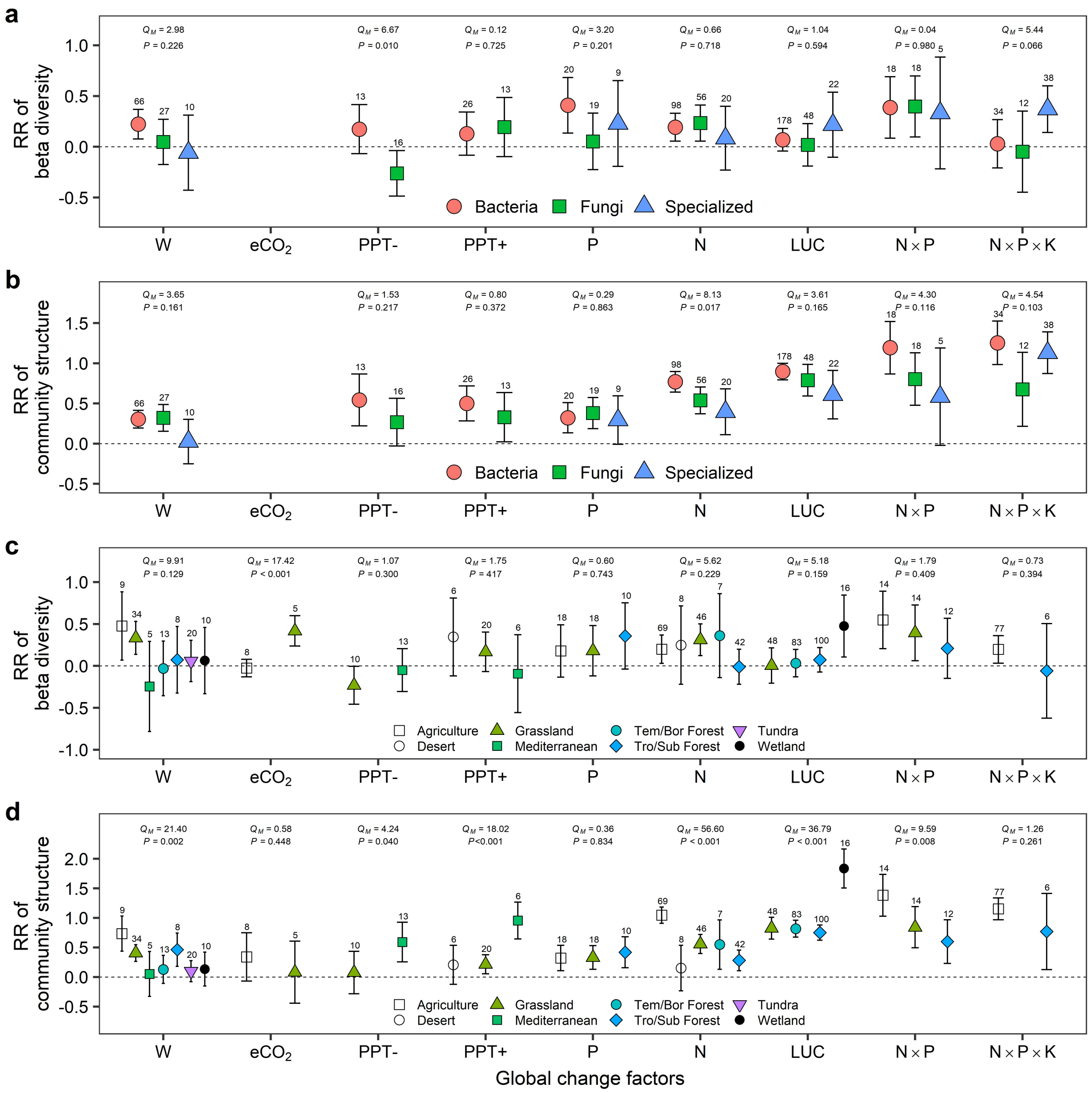

Supplementary Fig. 8 Responses of microbial beta diversity and community structure to global change factors by microbial groups and biomes. **a** Response ratio (RR) of beta diversity across microbial groups. **b** RR of community structure across microbial groups. **c** RR of beta diversity across biomes. **d** RR of community structure across biomes. Weighted means and their 95% confidence intervals of RRs are given. The numbers at the top of the confidence intervals represent the sample sizes. The significances of microbial groups and biome types are tested by omnibus test (Q_M). W, warming; eCO_2, carbon dioxide enrichment; PPT-, decreased precipitation; PPT+, increased precipitation; P, phosphorous addition; N, nitrogen addition; LUC, land use change; W×eCO_2, warming plus carbon dioxide enrichment; N×PPT+, nitrogen addition plus increased precipitation; N×P, nitrogen plus phosphorous addition; N×P×K, nitrogen plus phosphorous plus potassium addition. Tem/Bor, temperate/ boreal. Tro/Sub, tropical/subtropical. Source data are provided as a Source Data file.

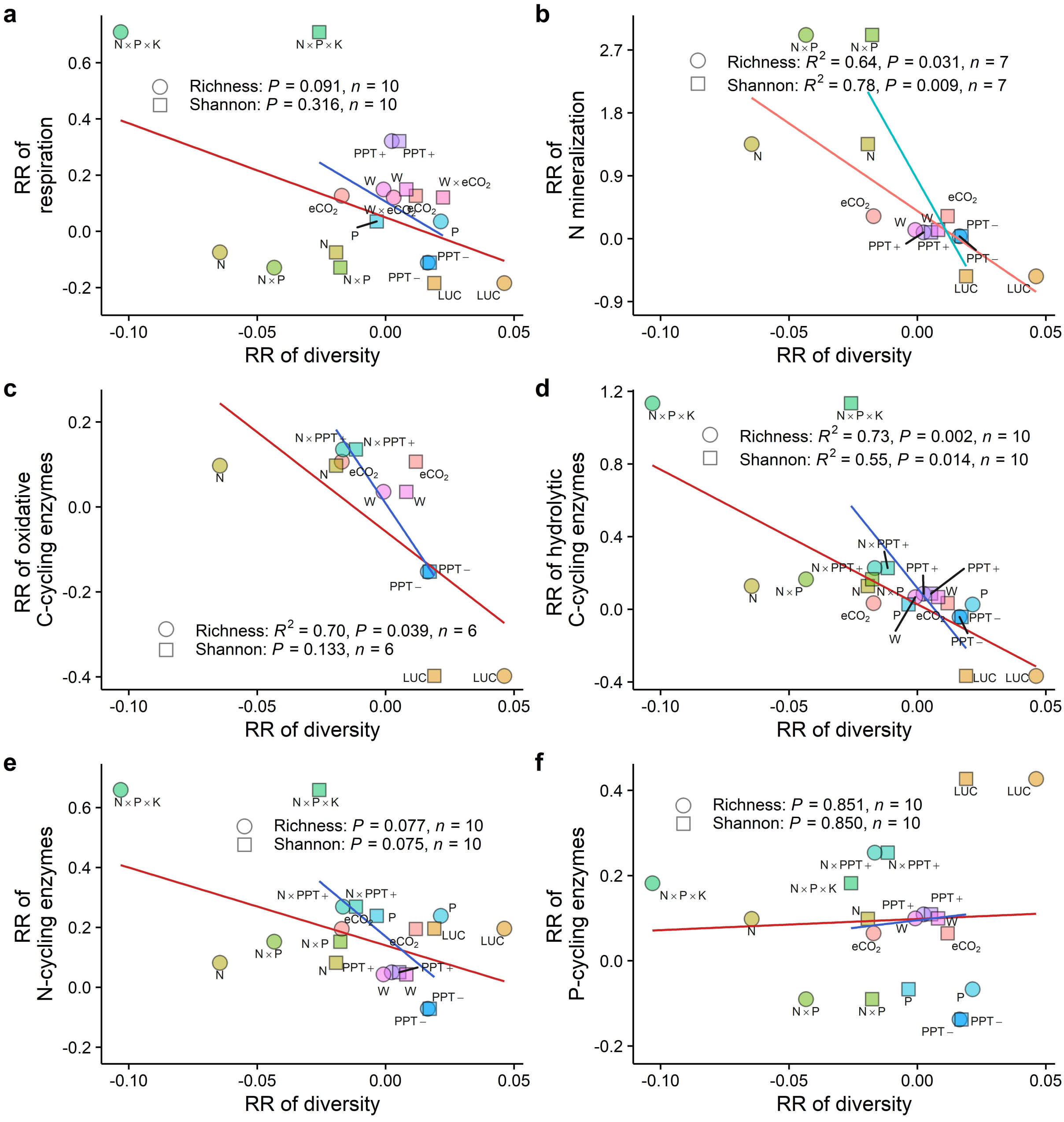

Supplementary Fig. 9 Linear relationships of the response ratios (RRs) between microbial alpha diversity response and different functional responses. **a** Linear relationship between RR of respiration and RR of richness or Shannon index (insignificant). **b** Linear relationship between RR of N mineralization and RR of richness ($y = -25.06x + 0.40$) or Shannon index ($y = -65.16x + 0.84$). **c** Linear relationship between RR of oxidative C-cycling enzymes and RR of richness ($y = -4.67x - 0.06$) or Shannon index (insignificant). **d** Linear relationship between RR of hydrolytic C-cycling enzymes and RR of richness ($y = -7.42x + 0.03$) or Shannon index ($y = -17.53x + 0.12$). **e** Linear relationship between RR of N-cycling enzymes and RR of richness or Shannon index (insignificant). **f** Linear relationship between RR of P-cycling enzymes and RR of richness or Shannon index (insignificant). Source data are provided as a Source Data file.

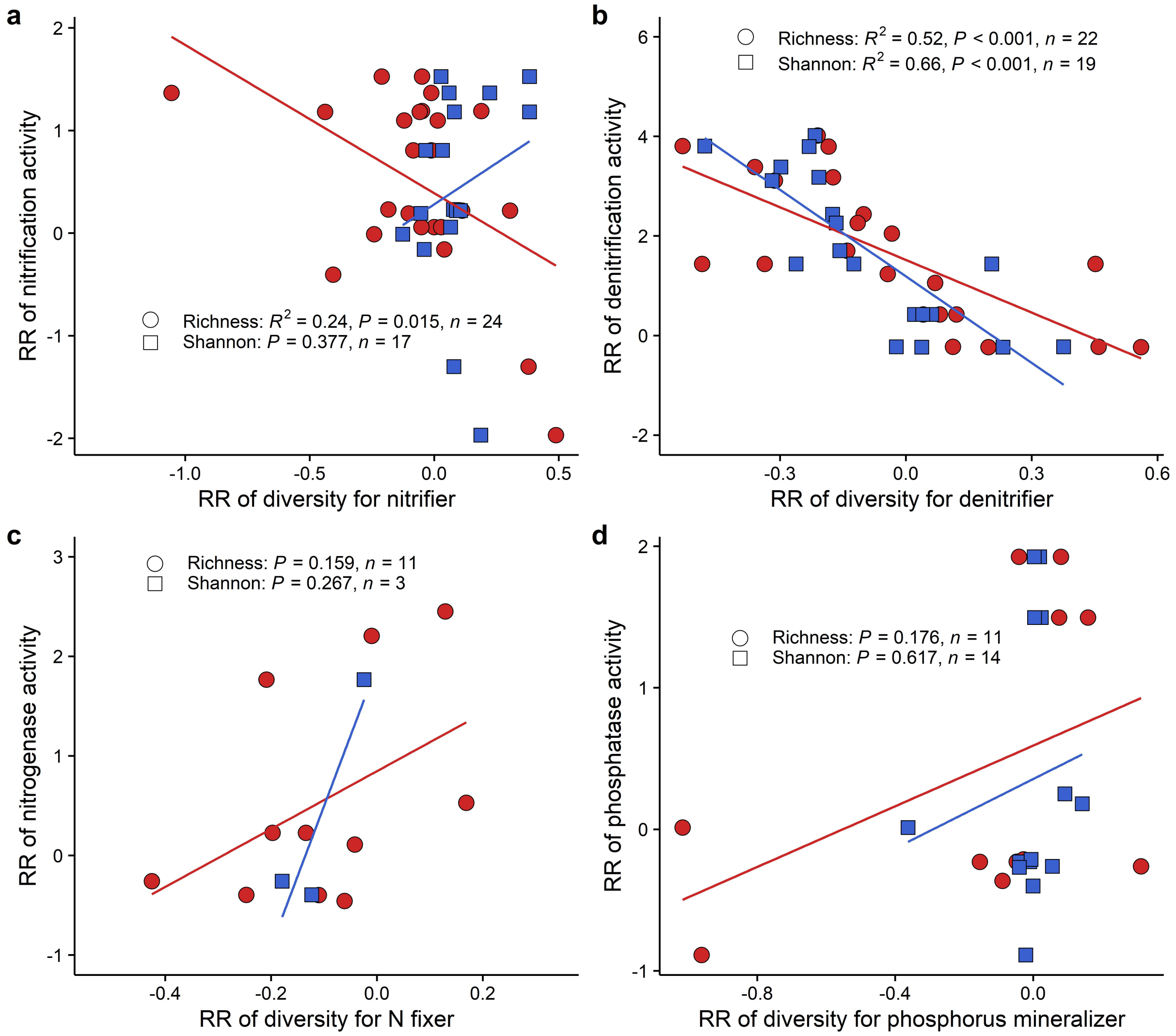

Supplementary Fig. 10 Coordinated changes between microbial alpha diversity and functions for specialized microbes. **a** Linear relationship between response ratio (RR) of nitrification activity and RRs of richness ($y = -1.44x + 0.39$) or Shannon index (insignificant) for nitrifier. **b** Linear relationship between RR of denitrification activity and RRs of richness ($y = -3.53x + 1.52$) or Shannon index ($y = -5.79x + 1.19$) for denitrifier. **c** Linear relationship between RR of nitrogenase activity and RRs of richness or Shannon index for nitrogen (N) fixer (insignificant). **d** Linear relationship between RR of phosphatase activity and RRs of richness or Shannon index for phosphorus mineralizer (insignificant). Source data are provided as a Source Data file.

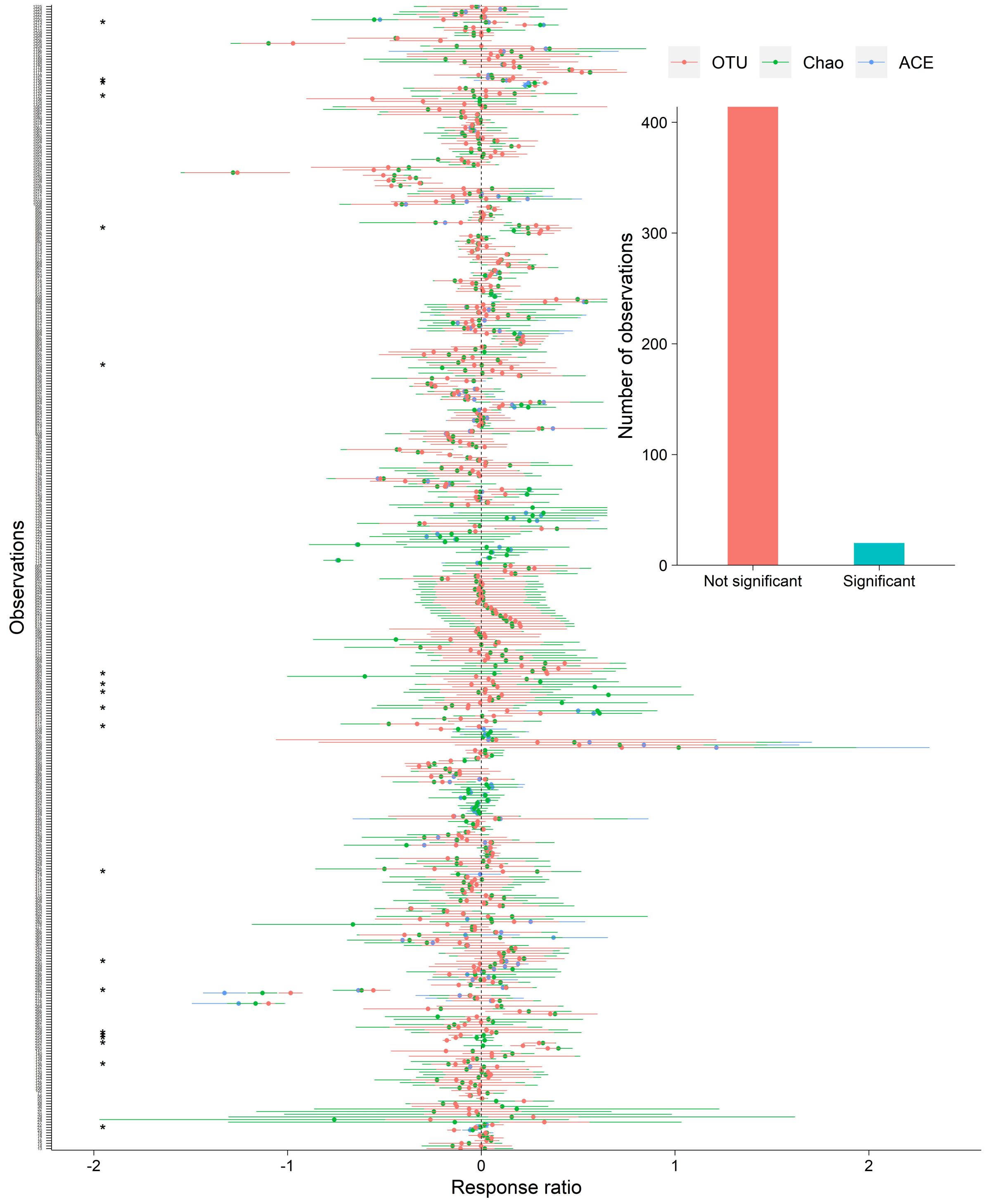

Supplementary Fig. 11 Comparisons of response ratios of different richness metrics. The asterisks on the left indicate significant differences among the two/three metrics based on omnibus test (Q_M). The inset bar plot shows the total number of significant *vs.* insignificant observations. Source data are provided as a Source Data file.

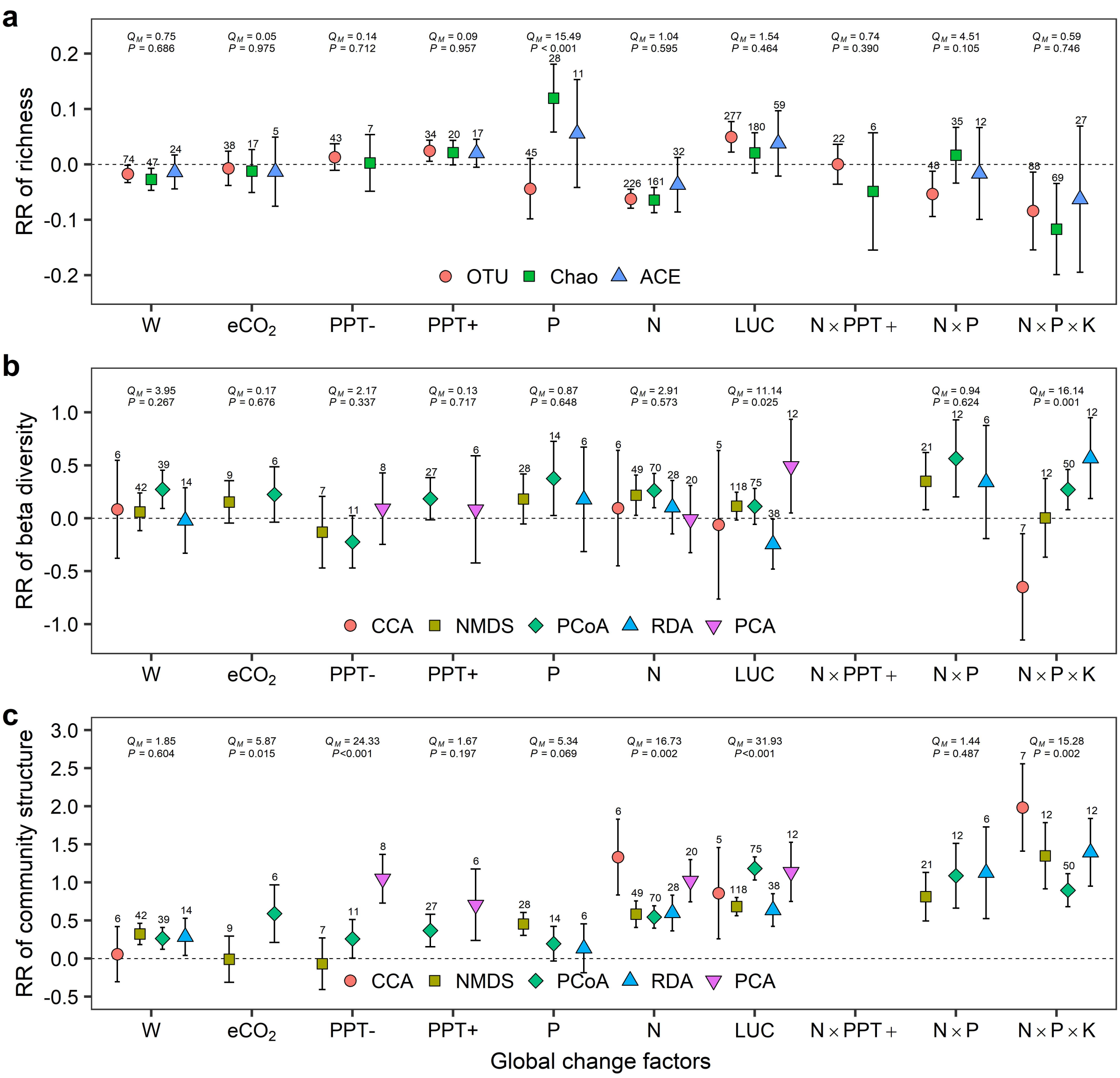

Supplementary Fig. 12 Comparisons of microbial responses to global change factors by different data analytical methods. **a** Response ratios (RRs) of microbial richness from different metrics. **b** RRs of microbial beta diversity from canonical correspondence analysis (CCA), non-metric multidimensional scaling (NMDS), principal correspondence analysis (PCoA), redundancy analysis (RDA), and principal component analysis (PCA) plots. **c** RRs of microbial community structure from CCA, NMDS, PCoA, RDA, and PCA plots. The significances of methods are tested by omnibus test (Q_M). Weighted means and their 95% confidence intervals of RRs are given. The numbers at the top of the confidence intervals represent the sample sizes. W, warming; eCO2, carbon dioxide enrichment; PPT-, decreased precipitation; PPT+, increased precipitation; P, phosphorous addition; N, nitrogen addition; LUC, land use change; W×eCO2, warming plus carbon dioxide enrichment; N×PPT+, nitrogen addition plus increased precipitation; N×P, nitrogen plus phosphorous addition; N×P×K, nitrogen plus phosphorous plus potassium addition. Source data are provided as a Source Data file.

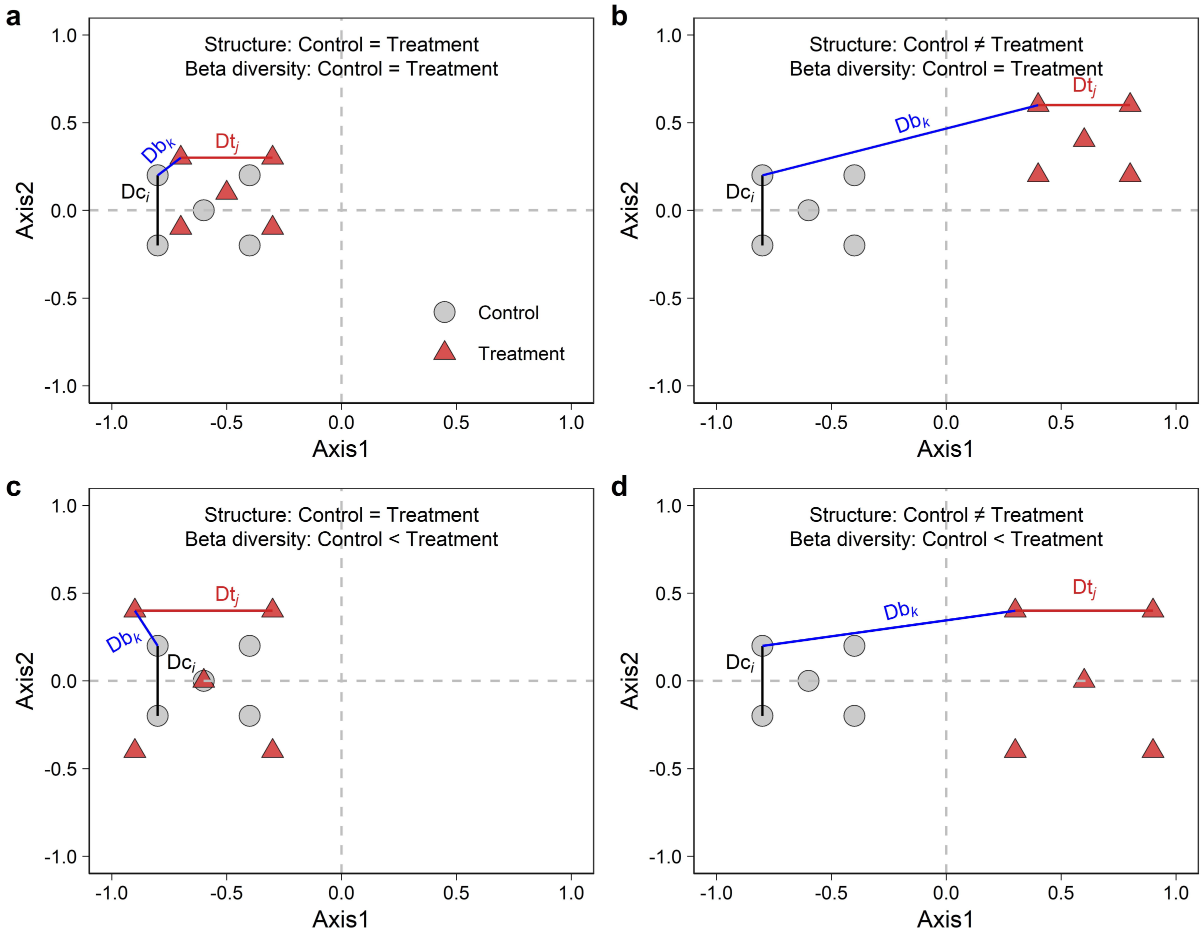

Supplementary Fig. 13 A diagrammatic sketch of the calculation of response of microbial community structure and beta diversity. Dc_i, the *i*th Euclidean distance within control, Dt_j, the *j*th Euclidean distance within treatment, Db_k, the *k*th Euclidean distance between control and treatment. The effect of global change factor on community composition is considered if the Euclidean distance between control and treatment is significantly greater than that within group. The effect of global change factor on beta diversity is considered if the Euclidean distance within treatment is significantly different from that within control.

Supplementary References 1

1 Acosta-Martínez, V., Dowd, S., Sun, Y. & Allen, V. Tag-encoded pyrosequencing analysis of bacterial diversity in a single soil type as affected by management and land use. *Soil Biol. Biochem.* **40**, 2762–2770 (2008).

2 Allen, K., Corre, M. D., Tjoa, A. & Veldkamp, E. Soil nitrogen-cycling responses to conversion of lowland forests to oil palm and rubber plantations in Sumatra, Indonesia. *PLoS ONE* **10**, e0133325 (2015).

3 Allison, S. D., McGuire, K. L. & Treseder, K. K. Resistance of microbial and soil properties to warming treatment seven years after boreal fire. *Soil Biol. Biochem.* **42**, 1872–1878 (2010).

4 Allison, S. D. & Treseder, K. K. Warming and drying suppress microbial activity and carbon cycling in boreal forest soils. *Glob. Change Biol.* **14**, 2898–2909 (2008).

5 Austin, E. E., Castro, H. F., Sides, K. E., Schadt, C. W. & Classen, A. T. Assessment of 10 years of CO_2 fumigation on soil microbial communities and function in a sweetgum plantation. *Soil Biol. Biochem.* **41**, 514–520 (2009).

6 Bastida, F. *et al.* Differential sensitivity of total and active soil microbial communities to drought and forest management. *Glob. Change Biol.* **23**, 4185–4203 (2017).

7 Berkelmann, D. *et al.* How rainforest conversion to agricultural systems in Sumatra (Indonesia) affects active soil bacterial communities. *Front. Microbiol.* **9**, 2381 (2018).

8 Birnbaum, C., Hopkins, A. J., Fontaine, J. B. & Enright, N. J. Soil fungal responses to experimental warming and drying in a Mediterranean shrubland. *Sci. Total Environ.* **683**, 524–536 (2019).

9 Bissett, A., Richardson, A. E., Baker, G. & Thrall, P. H. Long-term land use effects on soil microbial community structure and function. *Appl. Soil Ecol.* **51**, 66–78 (2011).

10 Brenzinger, K. *et al.* Soil conditions rather than long-term exposure to elevated CO_2 affect soil microbial communities associated with N-cycling. *Front. Microbiol.* **8**, 1976 (2017).

11 Brinkmann, N. *et al.* Intensive tropical land use massively shifts soil fungal communities. *Sci. Rep.* **9**, 1–11 (2019).

12 Bu, X. *et al.* Extreme drought slightly decreased soil labile organic C and N contents and altered microbial community structure in a subtropical evergreen forest. *For. Ecol. Manage.* **429**, 18–27 (2018).

13 Burton, A. J., Pregitzer, K. S., Crawford, J. N., Zogg, G. P. & Zak, D. R. Simulated chronic NO_3-deposition reduces soil respiration in northern hardwood forests. *Glob. Change Biol.* **10**, 1080–1091 (2004).

14 Butler, S. M. *et al.* Soil warming alters nitrogen cycling in a New England forest: implications for ecosystem function and structure. *Oecologia* **168**, 819–828 (2012).

15 Cai, Z. Q., Zhang, Y. H., Yang, C. & Wang, S. Land-use type strongly shapes community composition, but not always diversity of soil microbes in tropical China. *Catena* **165**, 369–380 (2018).

16 Camenzind, T. *et al.* Nitrogen and phosphorus additions impact arbuscular mycorrhizal abundance and molecular diversity in a tropical montane forest. *Glob. Change Biol.* **20**, 3646–3659 (2014).

17 Cao, C. *et al.* Land-use changes influence soil bacterial communities in a meadow grassland in Northeast China. *Solid Earth* **8**, 1119 (2017).

18 Cao, J. *et al.* Warming exerts a stronger effect than nitrogen addition on the soil arbuscular mycorrhizal fungal community in a young subtropical *Cunninghamia lanceolata* plantation. *Geoderma* **367**, 114273 (2020).

19 Carney, K. M., Hungate, B. A., Drake, B. G. & Megonigal, J. P. Altered soil microbial community at elevated CO_2 leads to loss of soil carbon. *Proc. Natl Acad. Sci. USA* **104**, 4990–4995 (2007).
20 Carney, K. M., Matson, P. A. & Bohannan, B. J. Diversity and composition of tropical soil nitrifiers across a plant diversity gradient and among land-use types. *Ecol. Lett.* **7**, 684–694 (2004).
21 Carson, C. M. & Zeglin, L. H. Long-term fire management history affects N-fertilization sensitivity, but not seasonality, of grassland soil microbial communities. *Soil Biol. Biochem.* **121**, 231–239 (2018).
22 Castañeda, L. E. & Barbosa, O. Metagenomic analysis exploring taxonomic and functional diversity of soil microbial communities in Chilean vineyards and surrounding native forests. *Peerj* **5**, e3098 (2017).
23 Castellano-Hinojosa, A., Correa-Galeote, D., González-López, J. & Bedmar, E. J. Effect of nitrogen fertilisers on nitrous oxide emission, nitrifier and denitrifier abundance and bacterial diversity in closed ecological systems. *Appl. Soil Ecol.* **145**, 103380 (2020).
24 Castro, H. F., Classen, A. T., Austin, E. E., Norby, R. J. & Schadt, C. W. Soil microbial community responses to multiple experimental climate change drivers. *Appl. Environ. Microbiol.* **76**, 999–1007 (2010).
25 Chang, E. H., Chen, T. H., Tian, G. L., Hsu, C. K. & Chiu, C. Y. Effect of 40 and 80 years of conifer regrowth on soil microbial activities and community structure in subtropical low mountain forests. *Forests* **7**, 244 (2016).
26 Che, R. *et al.* Total and active soil fungal community profiles were significantly altered by six years of warming but not by grazing. *Soil Biol. Biochem.* **139**, 107611 (2019).
27 Chen, C. *et al.* Microbial communities of an arable soil treated for 8 years with organic and inorganic fertilizers. *Biol. Fertil. Soils* **52**, 455–467 (2016).
28 Chen, D., Li, J., Lan, Z., Hu, S. & Bai, Y. Soil acidification exerts a greater control on soil respiration than soil nitrogen availability in grasslands subjected to long-term nitrogen enrichment. *Funct. Ecol.* **30**, 658–669 (2016).
29 Chen, D. M., Lan, Z. C., Hu, S. J. & Bai, Y. F. Effects of nitrogen enrichment on belowground communities in grassland: Relative role of soil nitrogen availability vs. soil acidification. *Soil Biol. Biochem.* **89**, 99–108 (2015).
30 Chen, J., Shen, W., Xu, H., Li, Y. & Luo, T. The composition of nitrogen-fixing microorganisms correlates with soil nitrogen content during reforestation: a comparison between legume and non-legume plantations. *Front. Microbiol.* **10**, 508 (2019).
31 Chen, Q., Niu, B., Hu, Y., Luo, T. & Zhang, G. Warming and increased precipitation indirectly affect the composition and turnover of labile-fraction soil organic matter by directly affecting vegetation and microorganisms. *Sci. Total Environ.* **714**, 136787 (2020).
32 Chen, W. *et al.* Consistent responses of surface-and subsurface soil fungal diversity to N enrichment are mediated differently by acidification and plant community in a semi-arid grassland. *Soil Biol. Biochem.* **127**, 110–119 (2018).
33 Chen, X. *et al.* Response of soil *phoD* phosphatase gene to long-term combined applications of chemical fertilizers and organic materials. *Appl. Soil Ecol.* **119**, 197–204 (2017).
34 Chen, X. *et al.* Impact of long-term phosphorus fertilizer inputs on bacterial *phoD* gene community in a maize field, Northeast China. *Sci. Total Environ.* **669**, 1011–1018 (2019).

35 Chen, X. *et al.* Soil alkaline phosphatase activity and bacterial *phoD* gene abundance and diversity under long-term nitrogen and manure inputs. *Geoderma* **349**, 36–44 (2019).
36 Chen, Y., Liu, J. & Liu, S. Effect of long-term mineral fertilizer application on soil enzyme activities and bacterial community composition. *Plant Soil Environ.* **64**, 571–577 (2018).
37 Chen, Z., Zhang, J., Xiong, Z., Pan, G. & Müller, C. Enhanced gross nitrogen transformation rates and nitrogen supply in paddy field under elevated atmospheric carbon dioxide and temperature. *Soil Biol. Biochem.* **94**, 80–87 (2016).
38 Colombo, F., Macdonald, C. A., Jeffries, T. C., Powell, J. R. & Singh, B. K. Impact of forest management practices on soil bacterial diversity and consequences for soil processes. *Soil Biol. Biochem.* **94**, 200–210 (2016).
39 Contosta, A. R., Frey, S. D. & Cooper, A. B. Seasonal dynamics of soil respiration and N mineralization in chronically warmed and fertilized soils. *Ecosphere* **2**, 1–21 (2011).
40 Coolon, J. D., Jones, K. L., Todd, T. C., Blair, J. M. & Herman, M. A. Long-term nitrogen amendment alters the diversity and assemblage of soil bacterial communities in tallgrass prairie. *PLoS ONE* **8**, e67884 (2013).
41 Corrales, A., Turner, B. L., Tedersoo, L., Anslan, S. & Dalling, J. W. Nitrogen addition alters ectomycorrhizal fungal communities and soil enzyme activities in a tropical montane forest. *Fungal Ecol.* **27**, 14–23 (2017).
42 Cuer, C. A. *et al.* Short-term effect of Eucalyptus plantations on soil microbial communities and soil-atmosphere methane and nitrous oxide exchange. *Sci. Rep.* **8**, 15133 (2018).
43 Cui, J., Wang, J., Xu, J., Xu, C. & Xu, X. Changes in soil bacterial communities in an evergreen broad-leaved forest in east China following 4 years of nitrogen addition. *J. Soils Sediments.* **17**, 2156–2164 (2017).
44 Cui, Y. *et al.* Responses of soil bacterial communities, enzyme activities, and nutrients to agricultural-to-natural ecosystem conversion in the Loess Plateau, China. *J. Soils Sediments.* **19**, 1427–1440 (2019).
45 Daquiado, A. R. *et al.* Pyrosequencing analysis of bacterial community diversity in long-term fertilized paddy field soil. *Appl. Soil Ecol.* **108**, 84–91 (2016).
46 de Menezes, A. B., Müller, C., Clipson, N. & Doyle, E. The soil microbiome at the Gi-FACE experiment responds to a moisture gradient but not to CO_2 enrichment. *Microbiology* **162**, 1572–1582 (2016).
47 DeAngelis, K. M. *et al.* Long-term forest soil warming alters microbial communities in temperate forest soils. *Front. Microbiol.* **6**, 104 (2015).
48 DeForest, J. L., Zak, D. R., Pregitzer, K. S. & Burton, A. J. Atmospheric nitrate deposition, microbial community composition, and enzyme activity in northern hardwood forests. *Soil Sci. Soc. Am. J.* **68**, 132–138 (2004).
49 Deng, J., Yin, Y., Zhu, W. & Zhou, Y. Variations in soil bacterial community diversity and structures among different revegetation types in the Baishilazi Nature Reserve. *Front. Microbiol.* **9**, 2874 (2018).
50 Deng, Q., Cheng, X., Bowatte, S., Newton, P. C. & Zhang, Q. Rhizospheric carbon-nitrogen interactions in a mixed-species pasture after 13 years of elevated CO_2. *Agric. Ecosyst. Environ.* **235**, 134–141 (2016).
51 Deng, Y. *et al.* Elevated carbon dioxide alters the structure of soil microbial communities. *Appl. Environ. Microbiol.* **78**, 2991–2995 (2012).

52 Deslippe, J. R., Hartmann, M., Simard, S. W. & Mohn, W. W. Long-term warming alters the composition of Arctic soil microbial communities. *FEMS Microbiol. Ecol.* **82**, 303–315 (2012).
53 Ding, J. *et al.* Effects of applying inorganic fertilizer and organic manure for 35 years on the structure and diversity of ammonia-oxidizing archaea communities in a Chinese Mollisols field. *MicrobiologyOpen*, e942 (2019).
54 Ding, J. L. *et al.* Effect of 35 years inorganic fertilizer and manure amendment on structure of bacterial and archaeal communities in black soil of northeast China. *Appl. Soil Ecol.* **105**, 187–195 (2016).
55 Dong, W. Y. *et al.* Responses of soil microbial communities and enzyme activities to nitrogen and phosphorus additions in Chinese fir plantations of subtropical China. *Biogeosciences* **12**, 5537–5546 (2015).
56 Drake, J. E. *et al.* Trenching reduces soil heterotrophic activity in a loblolly pine (*Pinus taeda*) forest exposed to elevated atmospheric [CO_2] and N fertilization. *Agric. For. Meteorol.* **165**, 43–52 (2012).
57 Du, Y. *et al.* Nitrogen fertilizer is a key factor affecting the soil chemical and microbial communities in a Mollisol. *Can. J. Microbiol.* **65**, 510–521 (2019).
58 Dunbar, J. *et al.* Common bacterial responses in six ecosystems exposed to 10 years of elevated atmospheric carbon dioxide. *Environ. Microbiol.* **14**, 1145–1158 (2012).
59 Eo, J. & Park, K. C. Long-term effects of imbalanced fertilization on the composition and diversity of soil bacterial community. *Agric. Ecosyst. Environ.* **231**, 176–182 (2016).
60 Fan, F. *et al.* Microbial mechanisms of the contrast residue decomposition and priming effect in soils with different organic and chemical fertilization histories. *Soil Biol. Biochem.* **135**, 213–221 (2019).
61 Fang, Y., Wang, F., Jia, X. & Chen, J. Distinct responses of ammonia-oxidizing bacteria and archaea to green manure combined with reduced chemical fertilizer in a paddy soil. *J. Soils Sediments* **19**, 1613–1623 (2019).
62 Felsmann, K. *et al.* Soil bacterial community structure responses to precipitation reduction and forest management in forest ecosystems across Germany. *PLoS ONE* **10**, e0122539 (2015).
63 Feng, J. *et al.* Long-term warming in Alaska enlarges the diazotrophic community in deep soils. *mBio* **10**, e02521–02518 (2019).
64 Finzi, A. C., Sinsabaugh, R. L., Long, T. M. & Osgood, M. P. Microbial community responses to atmospheric carbon dioxide enrichment in a warm-temperate forest. *Ecosystems* **9**, 215–226 (2006).
65 Francioli, D. *et al.* Mineral vs. organic amendments: microbial community structure, activity and abundance of agriculturally relevant microbes are driven by long-term fertilization strategies. *Front. Microbiol.* **7**, 1446 (2016).
66 Freedman, Z. B., Romanowicz, K. J., Upchurch, R. A. & Zak, D. R. Differential responses of total and active soil microbial communities to long-term experimental N deposition. *Soil Biol. Biochem.* **90**, 275–282 (2015).
67 Frey, S., Drijber, R., Smith, H. & Melillo, J. Microbial biomass, functional capacity, and community structure after 12 years of soil warming. *Soil Biol. Biochem.* **40**, 2904–2907 (2008).
68 Frey, S. D., Knorr, M., Parrent, J. L. & Simpson, R. T. Chronic nitrogen enrichment affects the structure and function of the soil microbial community in temperate hardwood and pine forests. *For. Ecol. Manage.* **196**, 159–171 (2004).
69 Frey, S. D., Lee, J., Melillo, J. M. & Six, J. The temperature response of soil microbial efficiency and its feedback to climate. *Nat. Clim. Change* **3**, 395 (2013).

70 Gao, J. *Effects of simulated warming and nitrogen deposition on soil microbes and enzyme activities in Chinese Fir in mid-subtropical*, Fujiang Normal university (2016).
71 Ge, Y., Chen, C., Xu, Z., Oren, R. & He, J. Z. The spatial factor, rather than elevated CO_2, controls the soil bacterial community in a temperate forest ecosystem. *Appl. Environ. Microbiol.* **76**, 7429–7436 (2010).
72 Gellie, N. J., Mills, J. G., Breed, M. F. & Lowe, A. J. Revegetation rewilds the soil bacterial microbiome of an old field. *Mol. Ecol.* **26**, 2895–2904 (2017).
73 Geml, J. *et al.* Long-term warming alters richness and composition of taxonomic and functional groups of arctic fungi. *FEMS Microbiol. Ecol.* **91**, fiv095 (2015).
74 Griffiths, B. S., Spilles, A. & Bonkowski, M. C:N:P stoichiometry and nutrient limitation of the soil microbial biomass in a grazed grassland site under experimental P limitation or excess. *Ecol. Process* **1**, 6 (2012).
75 Guenet, B. *et al.* The impact of long-term CO_2 enrichment and moisture levels on soil microbial community structure and enzyme activities. *Geoderma* **170**, 331–336 (2012).
76 Han, S. *et al.* Nitrite-oxidizing bacteria community composition and diversity are influenced by fertilizer regimes, but are independent of the soil aggregate in acidic subtropical red soil. *Front. Microbiol.* **9**, 885 (2018).
77 Hao, Y. Q. *et al.* Effects of nitrogen deposition on diversity and composition of soil bacterial community in a subtropical *Cunninghamia lanceolata* plantation. *Chinese J. Appl. Ecol.* **29**, 53–58 (2018).
78 Hartmann, M. *et al.* A decade of irrigation transforms the soil microbiome of a semi-arid pine forest. *Mol. Ecol.* **26**, 1190–1206 (2017).
79 Hayashi, K. *et al.* Free-air CO_2 enrichment (FACE) net nitrogen fixation experiment at a paddy soil surface under submerged conditions. *Nutr. Cycl. Agroecosystems* **98**, 57–69 (2014).
80 Hayden, H. L. *et al.* Changes in the microbial community structure of bacteria, archaea and fungi in response to elevated CO_2 and warming in an Australian native grassland soil. *Environ. Microbiol.* **14**, 3081–3096 (2012).
81 He, D. *et al.* Composition of the soil fungal community is more sensitive to phosphorus than nitrogen addition in the alpine meadow on the Qinghai-Tibetan Plateau. *Biol. Fertil. Soils* **52**, 1059–1072 (2016).
82 He, Z. *et al.* The phylogenetic composition and structure of soil microbial communities shifts in response to elevated carbon dioxide. *ISME J.* **6**, 259 (2012).
83 He, Z. *et al.* Distinct responses of soil microbial communities to elevated CO_2 and O_3 in a soybean agro-ecosystem. *ISME J.* **8**, 714–726 (2014).
84 He, Z. *et al.* Metagenomic analysis reveals a marked divergence in the structure of belowground microbial communities at elevated CO_2. *Ecol. Lett.* **13**, 564–575 (2010).
85 Hedo de Santiago, J., Lucas-Borja, M. E., Wic-Baena, C., Andrés-Abellán, M. & de las Heras, J. Effects of thinning and induced drought on microbiological soil properties and plant species diversity at dry and semiarid locations. *Land Degrad. Dev.* **27**, 1151–1162 (2016).
86 Henry, H. A. L., Juarez, J. D., Field, C. B. & Vitousek, P. M. Interactive effects of elevated CO_2, N deposition and climate change on extracellular enzyme activity and soil density fractionation in a California annual grassland. *Glob. Change Biol.* **11**, 1808–1815 (2005).
87 Holmes, W. E., Zak, D. R., Pregitzer, K. S. & King, J. S. Soil nitrogen transformations under *Populus tremuloides*, *Betula papyrifera* and *Acer saccharum* following 3 years exposure to elevated CO_2 and O_3. *Glob. Change Biol.* **9**, 1743–1750 (2003).

88 Homeier, J. *et al.* Tropical Andean forests are highly susceptible to nutrient inputs-Rapid effects of experimental N and P addition to an Ecuadorian montane forest. *PLoS ONE* **7**, e47128 (2012).
89 Hou, X. *et al.* Changes in soil physico-chemical properties following vegetation restoration mediate bacterial community composition and diversity in Changting, China. *Ecol. Eng.* **138**, 171–179 (2019).
90 Hovenden, M. J., Newton, P. C. & Osanai, Y. Warming has a larger and more persistent effect than elevated CO_2 on growing season soil nitrogen availability in a species-rich grassland. *Plant Soil* **421**, 417–428 (2017).
91 Hu, H. W. *et al.* Water addition regulates the metabolic activity of ammonia oxidizers responding to environmental perturbations in dry subhumid ecosystems. *Environ. Microbiol.* **17**, 444–461 (2015).
92 Hu, J., Lin, X., Bentivenga, S. P., Hou, X. Y. & Ji, B. Intraradical and extraradical communities of AM fungi associated with alfalfa respond differently to long-term phosphorus fertilization. *Flora* **258**, 151424 (2019).
93 Hu, J. L. *et al.* Population size and specific potential of P-mineralizing and -solubilizing bacteria under long-term P-deficiency fertilization in a sandy loam soil. *Pedobiologia* **53**, 49–58 (2009).
94 Hu, X. *et al.* Long-term application of nitrogen, not phosphate or potassium, significantly alters the diazotrophic community compositions and structures in a Mollisol in northeast China. *Res. Microbiol.* **170**, 147–155 (2019).
95 Hu, X. *et al.* Effects of over 30-year of different fertilization regimes on fungal community compositions in the black soils of northeast China. *Agric. Ecosyst. Environ.* **248**, 113–122 (2017).
96 Hu, X. *et al.* Chronic effects of different fertilization regimes on *nirS*-type denitrifier communities across the black soil region of Northeast China. *Pedosphere* **30**, 73–86 (2020).
97 Hu, Z. K. *et al.* Responses of rice paddy micro-food webs to elevated CO_2 are modulated by nitrogen fertilization and crop cultivars. *Soil Biol. Biochem.* **114**, 104–113 (2017).
98 Huang, G., Li, L., Su, Y. G. & Li, Y. Differential seasonal effects of water addition and nitrogen fertilization on microbial biomass and diversity in a temperate desert. *Catena* **161**, 27–36 (2018).
99 Huang, G., Li, Y. & Su, Y. G. Divergent responses of soil microbial communities to water and nitrogen addition in a temperate desert. *Geoderma* **251**, 55–64 (2015).
100 Huang, J. *et al.* Responses of soil nitrogen fixation to *Spartina alterniflora* invasion and nitrogen addition in a Chinese salt marsh. *Sci. Rep.* **6**, 20384 (2016).
101 Huang, L. *et al.* Soil bacterial community structure and extracellular enzyme activities under different land use types in a long-term reclaimed wetland. *J. Soils Sediments* **19**, 2543–2557 (2019).
102 Huang, Q., Wang, J., Wang, C. & Wang, Q. The 19-years inorganic fertilization increased bacterial diversity and altered bacterial community composition and potential functions in a paddy soil. *Appl. Soil Ecol.* **144**, 60–67 (2019).
103 Huang, R. *et al.* Nitrous oxide emission and the related denitrifier community: A short-term response to organic manure substituting chemical fertilizer. *Ecotox. Environ. Safe.* **192**, 110291 (2020).
104 Iversen, C. M., Keller, J. K., Garten Jr, C. T. & Norby, R. J. Soil carbon and nitrogen cycling and storage throughout the soil profile in a sweetgum plantation after 11 years of CO_2-enrichment. *Glob. Change Biol.* **18**, 1684–1697 (2012).

105 Jangid, K. *et al.* Development of soil microbial communities during tallgrass prairie restoration. *Soil Biol. Biochem.* **42**, 302–312 (2010).

106 Jangid, K. *et al.* Relative impacts of land-use, management intensity and fertilization upon soil microbial community structure in agricultural systems. *Soil Biol. Biochem.* **40**, 2843–2853 (2008).

107 Jangid, K. *et al.* Land-use history has a stronger impact on soil microbial community composition than aboveground vegetation and soil properties. *Soil Biol. Biochem.* **43**, 2184–2193 (2011).

108 Jin, V. & Evans, R. Elevated CO_2 increases microbial carbon substrate use and nitrogen cycling in Mojave Desert soils. *Glob. Change Biol.* **13**, 452–465 (2007).

109 Jin, X. *et al.* Soil bacterial and fungal communities and the associated nutrient cycling responses to forest conversion after selective logging in a subtropical forest of China. *For. Ecol. Manage.* **444**, 308–317 (2019).

110 Jing, X. *et al.* Neutral effect of nitrogen addition and negative effect of phosphorus addition on topsoil extracellular enzymatic activities in an alpine grassland ecosystem. *Appl. Soil Ecol.* **107**, 205–213 (2016).

111 Joa, J. H., Weon, H. Y., Hyun, H. N., Jeun, Y. C. & Koh, S. W. Effect of long-term different fertilization on bacterial community structures and diversity in Citrus orchard soil of volcanic ash. *J. Microbiol.* **52**, 995–1001 (2014).

112 Jumpponen, A. & Jones, K. L. Tallgrass prairie soil fungal communities are resilient to climate change. *Fungal Ecol.* **10**, 44–57 (2014).

113 Jung, J. *et al.* Responses of surface SOC to long-term experimental warming vary between different heath types in the High Arctic tundra. *Eur. J. Soil Sci.* (2019).

114 Kardol, P., Cregger, M. A., Campany, C. E. & Classen, A. T. Soil ecosystem functioning under climate change: plant species and community effects. *Ecology* **91**, 767–781 (2010).

115 Kerfahi, D. *et al.* Distinctive soil archaeal communities in different variants of tropical equatorial forest. *Microb. Ecol.* **76**, 215–225 (2018).

116 Khan, M. W. *et al.* Deforestation impacts network co-occurrence patterns of microbial communities in Amazon soils. *FEMS Microbiol. Ecol.* **95**, fiy230 (2018).

117 Kim, D. *et al.* Passive warming effect on soil microbial community and humic substance degradation in maritime Antarctic region. *J. Basic Microb.* **58**, 513–522 (2018).

118 Koehler, B., Corre, M. D., Veldkamp, E., Wullaert, H. & Wright, S. J. Immediate and long-term nitrogen oxide emissions from tropical forest soils exposed to elevated nitrogen input. *Glob. Change Biol.* **15**, 2049–2066 (2009).

119 Kong, Y. *et al.* Long-term fertilization regimes change soil nitrification potential by impacting active autotrophic ammonia oxidizers and nitrite oxidizers as assessed by DNA stable isotope probing. *Environ. Microbiol.* **21,** 1224–1240 (2019).

120 Koyama, A., Wallenstein, M. D., Simpson, R. T. & Moore, J. C. Carbon-degrading enzyme activities stimulated by increased nutrient availability in arctic tundra soils. *PLoS ONE* **8**, e77212 (2013).

121 Koyama, A., Wallenstein, M. D., Simpson, R. T. & Moore, J. C. Soil bacterial community composition altered by increased nutrient availability in Arctic tundra soils. *Front. Microbiol.* **5**, 516 (2014).

122 Kuffner, M. *et al.* Effects of season and experimental warming on the bacterial community in a temperate mountain forest soil assessed by 16S rRNA gene pyrosequencing. *FEMS Microbiol. Ecol.* **82**, 551–562 (2012).

123 Kuramae, E. E. *et al.* Microbial secondary succession in a chronosequence of chalk grasslands. *ISME J.* **4**, 711–715 (2010).

124 Kuramae, E. E. *et al.* Soil characteristics more strongly influence soil bacterial communities than land-use type. *FEMS Microbiol. Ecol.* **79**, 12–24 (2012).
125 Lan, G., Li, Y., Wu, Z. & Xie, G. Soil bacterial diversity impacted by conversion of secondary forest to rubber or eucalyptus plantations: a case study of Hainan Island, South China. *For. Sci.* **63**, 87–93 (2017).
126 Lang, M., Christie, P., Zhang, J. & Li, X. Long-term phosphorus application to a maize monoculture influences the soil microbial community and its feedback effects on maize seedling biomass. *Appl. Soil Ecol.* **128**, 12–22 (2018).
127 Larson, J. L., Zak, D. R. & Sinsabaugh, R. L. Extracellular enzyme activity beneath temperate trees growing under elevated carbon dioxide and ozone. *Soil Sci. Soc. Am. J.* **66**, 1848–1856 (2002).
128 Lauber, C. L., Strickland, M. S., Bradford, M. A. & Fierer, N. The influence of soil properties on the structure of bacterial and fungal communities across land-use types. *Soil Biol. Biochem.* **40**, 2407–2415 (2008).
129 Lee, S. H. & Kang, H. Elevated CO_2 causes a change in microbial communities of rhizosphere and bulk soil of salt marsh system. *Appl. Soil Ecol.* **108**, 307–314 (2016).
130 Lee, S. H., Megonigal, P. J., Langley, A. J. & Kang, H. Elevated CO_2 and nitrogen addition affect the microbial abundance but not the community structure in salt marsh ecosystem. *Appl. Soil Ecol.* **117**, 129–136 (2017).
131 Lesaulnier, C. *et al.* Elevated atmospheric CO_2 affects soil microbial diversity associated with trembling aspen. *Environ. Microbiol.* **10**, 926–941 (2008).
132 Li, C. H., Tang, L. S., Jia, Z. J. & Li, Y. Profile changes in the soil microbial community when desert becomes oasis. *PLoS ONE* **10**, e0139626 (2015).
133 Li, F. R., Liu, J. L., Ren, W. & Liu, L. L. Land-use change alters patterns of soil biodiversity in arid lands of northwestern China. *Plant Soil* **428**, 371–388 (2018).
134 Li, G. *et al.* Precipitation affects soil microbial and extracellular enzymatic responses to warming. *Soil Biol. Biochem.* **120**, 212–221 (2018).
135 Li, G., Kim, S., Han, S. H., Chang, H. & Son, Y. Effect of soil moisture on the response of soil respiration to open-field experimental warming and precipitation manipulation. *Forests* **8**, 56 (2017).
136 Li, H. *et al.* Responses of soil bacterial communities to nitrogen deposition and precipitation increment are closely linked with aboveground community variation. *Microb. Ecol.* **71**, 974–989 (2016).
137 Li, H. *et al.* Responses of soil microbial functional genes to global changes are indirectly influenced by aboveground plant biomass variation. *Soil Biol. Biochem.* **104**, 18–29 (2017).
138 Li, J. *Response of soil hydrolase activity to simulated nitrogen deposition and increased precipitation in a Stipa baicalensis meadow steppe,* Northeast Normal University (2015).
139 Li, J. G. *et al.* Effect of different levels of nitrogen on rhizosphere bacterial community structure in intensive monoculture of greenhouse lettuce. *Sci. Rep.* **6**, 25305 (2016).
140 Li, L. *et al.* Different responses of absorptive roots and arbuscular mycorrhizal fungi to fertilization provide diverse nutrient acquisition strategies in Chinese fir. *For. Ecol. Manage.* **433**, 64–72 (2019).
141 Li, P., Shen, C., Jiang, L., Feng, Z. & Fang, J. Difference in soil bacterial community composition depends on forest type rather than nitrogen and phosphorus additions in tropical montane rainforests. *Biol. Fertil. Soils* **55**, 313–323 (2019).
142 Li, Q. *et al.* Nitrogen depositions increase soil respiration and decrease temperature sensitivity in a Moso bamboo forest. *Agric. For. Meteorol.* **268**, 48–54 (2019).

143 Li, Q., Song, X., Gu, H. & Gao, F. Nitrogen deposition and management practices increase soil microbial biomass carbon but decrease diversity in Moso bamboo plantations. *Sci. Rep.* **6**, 28235 (2016).
144 Li, S. *Effects of rainfall change on soil microbial characteristics in Korean pine broad-leaved forest.* Northeast Forestry University (2011).
145 Li, W., Sheng, H., Ekawati, D., Jiang, Y. & Yang, H. Variations in the compositions of soil bacterial and fungal communities due to microhabitat effects induced by simulated nitrogen deposition of a Bamboo Forest in Wetland. *Forests* **10**, 1098 (2019).
146 Li, Y. *et al.* Soil bacterial community responses to warming and grazing in a Tibetan alpine meadow. *FEMS Microbiol. Ecol.* **92**, fiv152 (2016).
147 Li, Y., Liu, Y., Wu, S., Niu, L. & Tian, Y. Microbial properties explain temporal variation in soil respiration in a grassland subjected to nitrogen addition. *Sci. Rep.* **5**, 18496 (2015).
148 Li, Y., Nie, C., Liu, Y., Du, W. & He, P. Soil microbial community composition closely associates with specific enzyme activities and soil carbon chemistry in a long-term nitrogen fertilized grassland. *Sci. Total Environ.* **654**, 264–274 (2019).
149 Li, Y. *et al.* Differential mechanisms underlying responses of soil bacterial and fungal communities to nitrogen and phosphorus inputs in a subtropical forest. *Peerj* **7**, e7631 (2019).
150 Li, Y. *et al.* Elevated CO_2 increases nitrogen fixation at the reproductive phase contributing to various yield responses of soybean cultivars. *Front. Plant Sci.* **8**, 1546 (2017).
151 Liao, H., Li, Y. & Yao, H. Fertilization with inorganic and organic nutrients changes diazotroph community composition and N-fixation rates. *J. Soils Sediments* **18**, 1076–1086 (2018).
152 Lin, X. G. *et al.* Long-term balanced fertilization decreases arbuscular mycorrhizal fungal diversity in an arable soil in north China revealed by 454 pyrosequencing. *Environ. Sci. Technol.* **46**, 5764–5771 (2012).
153 Lin, Y. T., Whitman, W. B., Coleman, D. C. & Chiu, C. Y. Effects of reforestation on the structure and diversity of bacterial communities in subtropical low mountain forest soils. *Front. Microbiol.* **9**, 1968 (2018).
154 Ling, N. *et al.* Differential responses of soil bacterial communities to long-term N and P inputs in a semi-arid steppe. *Geoderma* **292**, 25–33 (2017).
155 Lipson, D. A., Blair, M., Barron-Gafford, G., Grieve, K. & Murthy, R. Relationships between microbial community structure and soil processes under elevated atmospheric carbon dioxide. *Microb. Ecol.* **51**, 302–314 (2006).
156 Lipson, D. A., Kuske, C. R., Gallegos-Graves, L. V. & Oechel, W. C. Elevated atmospheric CO_2 stimulates soil fungal diversity through increased fine root production in a semiarid shrubland ecosystem. *Glob. Change Biol.* **20**, 2555–2565 (2014).
157 Lipson, D. A., Wilson, R. F. & Oechel, W. C. Effects of elevated atmospheric CO_2 on soil microbial biomass, activity, and diversity in a chaparral ecosystem. *Appl. Environ. Microbiol.* **71**, 8573–8580 (2005).
158 Liu, C. *et al.* Linkages between nutrient ratio and the microbial community in rhizosphere soil following fertilizer management. *Environ. Res.* **184**, 109261 (2020).
159 Liu, D. *et al.* Microbial functionality as affected by experimental warming of a temperate mountain forest soil-A metaproteomics survey. *Appl. Soil Ecol.* **117**, 196–202 (2017).

160 Liu, L. *et al.* Interactive effects of nitrogen and phosphorus on soil microbial communities in a tropical forest. *PLoS ONE* **8**, e61188 (2013).
161 Liu, S. E., Wang, H., Deng, Y., Tian, P. & Wang, Q. K. Forest conversion induces seasonal variation in microbial beta-diversity. *Environ. Microbiol.* **20**, 111–123 (2018).
162 Liu, X. *Soil fungal community structure and diversity in response to nitrogen addition and increased precipitation in a Stipa baicalensis meadow steppe*, Northeast Normal University, (2016).
163 Liu, X. *et al.* Will nitrogen deposition mitigate warming-increased soil respiration in a young subtropical plantation? *Agric. For. Meteorol.* **246**, 78–85 (2017).
164 Liu, Y. *et al.* Short-term responses of microbial community and functioning to experimental CO_2 enrichment and warming in a Chinese paddy field. *Soil Biol. Biochem.* **77**, 58–68 (2014).
165 Liu, Y. *et al.* Abundance and composition response of wheat field soil bacterial and fungal communities to elevated CO_2 and increased air temperature. *Biol. Fertil. Soils* **53**, 3–8 (2017).
166 Lu, M. *et al.* Contribution of soil variables to bacterial community composition following land use change in Napahai plateau wetlands. *J. Environ. Manage.* **246**, 77–84 (2019).
167 Luo, C. *et al.* Soil microbial community responses to a decade of warming as revealed by comparative metagenomics. *Appl. Environ. Microbiol.* **80**, 1777–1786 (2014).
168 Luo, G. *et al.* Long-term fertilisation regimes affect the composition of the alkaline phosphomonoesterase encoding microbial community of a vertisol and its derivative soil fractions. *Biol. Fertil. Soils* **53**, 375–388 (2017).
169 Luo, G. *et al.* Understanding how long-term organic amendments increase soil phosphatase activities: Insight into *phoD*- and *phoC*-harboring functional microbial populations. *Soil Biol. Biochem.* **139**, 107632 (2019).
170 Lupatini, M. *et al.* Land-use change and soil type are drivers of fungal and archaeal communities in the Pampa biome. *World J. Microbiol. Biotechnol.* **29**, 223–233 (2013).
171 Ma, L. *et al.* Divergent responses of bacterial activity, structure, and co-occurrence patterns to long-term unbalanced fertilization without nitrogen, phosphorus, or potassium in a cultivated vertisol. *Environ. Sci. Pollut. Res.* **26**, 12741–12754 (2019).
172 Ma, M. *et al.* Effect of long-term fertilization strategies on bacterial community composition in a 35-year field experiment of Chinese Mollisols. *AMB Express* **8**, 20 (2018).
173 Ma, S. *et al.* Plant and soil microbe responses to light, warming and nitrogen addition in a temperate forest. *Funct. Ecol.* **32**, 1293–1303 (2018).
174 Maček, I. *et al.* Impacts of long-term elevated atmospheric CO_2 concentrations on communities of arbuscular mycorrhizal fungi. *Mol. Ecol.* **28**, 3445–3458 (2019).
175 Mackelprang, R. *et al.* Microbial community structure and functional potential in cultivated and native tallgrass prairie soils of the midwestern United States. *Front. Microbiol.* **9**, 1775 (2018).
176 Maitra, P. *et al.* Effect of drought and season on arbuscular mycorrhizal fungi in a subtropical secondary forest. *Fungal Ecol.* **41**, 107–115 (2019).
177 Manpoong, C. *et al.* Linking rhizosphere soil biochemical and microbial community characteristics across different land use systems in mountainous region in Northeast India. *Meta Gene* **23**, 100625 (2020).
178 Marín, C. *et al.* Functional land-use change effects on soil fungal communities in Chilean temperate rainforests. *J. Soil Sci. Plant Nutr.* **17**, 985–1002 (2017).

179 Matamala, R. & Drake, B. G. The influence of atmospheric CO_2 enrichment on plant-soil nitrogen interactions in a wetland plant community on the Chesapeake Bay. *Plant Soil* **210**, 93–101 (1999).
180 Matulich, K. L. *Role of microbial communities in mediating an ecosystem's response to global change*, UC Irvine, (2015).
181 Matulich, K. L. *et al.* Temporal variation overshadows the response of leaf litter microbial communities to simulated global change. *ISME J.* **9**, 2477 (2015).
182 Mcgee, K. M., Eaton, W. D., Shokralla, S. & Hajibabaei, M. Determinants of soil bacterial and fungal community composition toward carbon-use efficiency across primary and secondary forests in a Costa Rican conservation area. *Microb. Ecol.* **77**, 148–167 (2019).
183 McHugh, T. A. *et al.* Bacterial, fungal, and plant communities exhibit no biomass or compositional response to two years of simulated nitrogen deposition in a semiarid grassland. *Environ. Microbiol.* **19**, 1600–1611 (2017).
184 Meng, M. *et al.* Impacts of forest conversion on soil bacterial community composition and diversity in subtropical forests. *Catena* **175**, 167–173 (2019).
185 Moora, M. *et al.* Anthropogenic land use shapes the composition and phylogenetic structure of soil arbuscular mycorrhizal fungal communities. *FEMS Microbiol. Ecol.* **90**, 609–621 (2014).
186 Morales, S. E., Trouche, B., Wakelin, S., Banabas, M. & Nelson, P. N. Shifts in prokaryotic communities under forest and grassland within a tropical mosaic landscape. *Appl. Soil Ecol.* **125**, 156–161 (2018).
187 Mueller, R. C., Belnap, J. & Kuske, C. R. Soil bacterial and fungal community responses to nitrogen addition across soil depth and microhabitat in an arid shrubland. *Front. Microbiol.* **6**, 891 (2015).
188 Müller, K., Deurer, M. & Newton, P. C. Is there a link between elevated atmospheric carbon dioxide concentration, soil water repellency and soil carbon mineralization? *Agric. Ecosyst. Environ.* **139**, 98–109 (2010).
189 Na, X. *et al.* Vegetation biomass and soil moisture coregulate bacterial community succession under altered precipitation regimes in a desert steppe in northwestern China. *Soil Biol. Biochem.* **136**, 107520 (2019).
190 Navarrete, A. A. *et al.* Soil microbiome responses to the short-term effects of Amazonian deforestation. *Mol. Ecol.* **24**, 2433–2448 (2015).
191 Newsham, K. K. *et al.* Bacterial community composition and diversity respond to nutrient amendment but not warming in a maritime Antarctic soil. *Microb. Ecol.* **78**, 974–984 (2019).
192 Nickel, U. T. *et al.* Quantitative losses vs. qualitative stability of ectomycorrhizal community responses to 3 years of experimental summer drought in a beech-spruce forest. *Glob. Change Biol.* **24** (2018).
193 Nie, Y. X. *et al.* Ammonium nitrogen content is a dominant predictor of bacterial community composition in an acidic forest soil with exogenous nitrogen enrichment. *Sci. Total Environ.* **624**, 407–415 (2018).
194 Nielsen, U. N. *et al.* Response of belowground communities to short-term phosphorus addition in a phosphorus-limited woodland. *Plant Soil* **391**, 321–331 (2015).
195 Niu, L. *et al.* Vegetation succession influences soil carbon sequestration in coastal alkali-saline soils in southeast China. *Sci. Rep.* **8**, 9728 (2018).
196 Ochoa-Hueso, R. *et al.* Drought consistently alters the composition of soil fungal and bacterial communities in grasslands from two continents. *Glob. Change Biol.* **24**, 2818–2827 (2018).

197 Okada, H. *et al.* Elevated temperature has stronger effects on the soil food web of a flooded paddy than does CO_2. *Soil Biol. Biochem.* **70**, 166–175 (2014).
198 Orgiazzi, A. *et al.* Unravelling soil fungal communities from different Mediterranean land-use backgrounds. *PLoS ONE* **7**, e34847 (2012).
199 Orwin, K., Dickie, I., Holdaway, R. & Wood, J. A comparison of the ability of PLFA and 16S rRNA gene metabarcoding to resolve soil community change and predict ecosystem functions. *Soil Biol. Biochem.* **117**, 27–35 (2018).
200 Osburn, E. D. *et al.* Soil bacterial and fungal communities exhibit distinct long-term responses to disturbance in temperate forests. *Front. Microbiol.* **10**, 2872 (2019).
201 Ouyang, Y. & Norton, J. M. Short-term nitrogen fertilization affects microbial community composition and nitrogen mineralization functions in an agricultural soil. *Appl. Environ. Microbiol.* **86** (2020).
202 Ouyang, Y., Norton, J. M., Stark, J. M., Reeve, J. R. & Habteselassie, M. Y. Ammonia-oxidizing bacteria are more responsive than archaea to nitrogen source in an agricultural soil. *Soil Biol. Biochem.* **96**, 4–15 (2016).
203 Panneerselvam, P. *et al.* Influence of elevated CO_2 on arbuscular mycorrhizal fungal community elucidated using Illumina MiSeq platform in sub-humid tropical paddy soil. *Appl. Soil Ecol.* **145**, 103344 (2020).
204 Pendall, E., Osanai, Y., Williams, A. L. & Hovenden, M. J. Soil carbon storage under simulated climate change is mediated by plant functional type. *Glob. Change Biol.* **17**, 505–514 (2011).
205 Peralta, A. L. & Wander, M. M. Soil organic matter dynamics under soybean exposed to elevated [CO_2]. *Plant Soil* **303**, 69–81 (2008).
206 Phillips, R. P., Finzi, A. C. & Bernhardt, E. S. Enhanced root exudation induces microbial feedbacks to N cycling in a pine forest under long-term CO_2 fumigation. *Ecol. Lett.* **14**, 187–194 (2011).
207 Pold, G. *et al.* Long-term warming alters carbohydrate degradation potential in temperate forest soils. *Appl. Environ. Microbiol.* **82**, 6518–6530 (2016).
208 Pregitzer, K. S., Burton, A. J., King, J. S. & Zak, D. R. Soil respiration, root biomass, and root turnover following long-term exposure of northern forests to elevated atmospheric CO_2 and tropospheric O_3. *New Phytol.* **180**, 153–161 (2008).
209 Qiu, J. *et al.* The influence of land use patterns on soil bacterial community structure in the Karst Graben Basin of Yunnan Province, China. *Forests* **11**, 51 (2020).
210 Ragot, S. A., Kertesz, M. A., Mészáros, É., Frossard, E. & Bünemann, E. K. Soil *phoD* and *phoX* alkaline phosphatase gene diversity responds to multiple environmental factors. *FEMS Microbiol. Ecol.* **93**, fiw212 (2017).
211 Ramirez, K. S., Lauber, C. L., Knight, R., Bradford, M. A. & Fierer, N. Consistent effects of nitrogen fertilization on soil bacterial communities in contrasting systems. *Ecology* **91**, 3463–3470 (2010).
212 Rampelotto, P. H., de Siqueira Ferreira, A., Barboza, A. D. M. & Roesch, L. F. W. Changes in diversity, abundance, and structure of soil bacterial communities in Brazilian Savanna under different land use systems. *Microb. Ecol.* **66**, 593–607 (2013).
213 Randall, K. *et al.* Soil bacterial community structure and functional responses across a long-term mineral phosphorus (Pi) fertilisation gradient differ in grazed and cut grasslands. *Appl. Soil Ecol.* **138**, 134–143 (2019).
214 Ren, F. *et al.* Contrasting effects of nitrogen and phosphorus addition on soil respiration in an alpine grassland on the Qinghai-Tibetan Plateau. *Sci. Rep.* **6**, 34786 (2016).

215 Ren, G. *et al.* Response of soil, leaf endosphere and phyllosphere bacterial communities to elevated CO_2 and soil temperature in a rice paddy. *Plant Soil* **392**, 27–44 (2015).
216 Rodrigues, J. L. *et al.* Conversion of the Amazon rainforest to agriculture results in biotic homogenization of soil bacterial communities. *Proc. Natl Acad. Sci. USA* **110**, 988–993 (2013).
217 Rustad, L. *et al.* A meta-analysis of the response of soil respiration, net nitrogen mineralization, and aboveground plant growth to experimental ecosystem warming. *Oecologia* **126**, 543–562 (2001).
218 Schindlbacher, A., Schnecker, J., Takriti, M., Borken, W. & Wanek, W. Microbial physiology and soil CO_2 efflux after 9 years of soil warming in a temperate forest–no indications for thermal adaptations. *Glob. Change Biol.* **21**, 4265–4277 (2015).
219 Schleuss, P. M. *et al.* Stoichiometric controls of soil carbon and nitrogen cycling after long-term nitrogen and phosphorus addition in a mesic grassland in South Africa. *Soil Biol. Biochem.* **135**, 294–303 (2019).
220 Schmidt, I. K., Jonasson, S., Shaver, G. R., Michelsen, A. & Nordin, A. Mineralization and distribution of nutrients in plants and microbes in four arctic ecosystems: responses to warming. *Plant Soil* **242**, 93–106 (2002).
221 Sengupta, A. & Dick, W. A. Methanotrophic bacterial diversity in two diverse soils under varying land-use practices as determined by high-throughput sequencing of the pmoA gene. *Appl. Soil Ecol.* **119**, 35–45 (2017).
222 Seuradge, B. J., Oelbermann, M. & Neufeld, J. D. Depth-dependent influence of different land-use systems on bacterial biogeography. *FEMS Microbiol. Ecol.* **93**, fiw239 (2017).
223 Shao, P. *et al.* Secondary successional forests undergo tightly-coupled changes in soil microbial community structure and soil organic matter. *Soil Biol. Biochem.* **128**, 56–65 (2019).
224 Shao, R., Deng, L., Yang, Q. & Shangguan, Z. Nitrogen fertilization increase soil carbon dioxide efflux of winter wheat field: A case study in Northwest China. *Soil Till. Res.* **143**, 164–171 (2014).
225 Shao, Y. *et al.* Plants mitigate detrimental nitrogen deposition effects on soil biodiversity. *Soil Biol. Biochem.* **127**, 178–186 (2018).
226 She, W. *et al.* Resource availability drives responses of soil microbial communities to short-term precipitation and nitrogen addition in a desert shrubland. *Front. Microbiol.* **9**, 186 (2018).
227 Sheik, C. S. *et al.* Effect of warming and drought on grassland microbial communities. *ISME J.* **5**, 1692–1700 (2011).
228 Sheldrake, M. *et al.* Responses of arbuscular mycorrhizal fungi to long-term inorganic and organic nutrient addition in a lowland tropical forest. *ISME J.* **12**, 2433–2445 (2018).
229 Shi, Y. *et al.* Soil fungal community assembly processes under long-term fertilization. *Eur. J. Soil Sci.* (2019).
230 Sinsabaugh, R. L. *et al.* Soil microbial responses to nitrogen addition in arid ecosystems. *Front. Microbiol.* **6**, 819 (2015).
231 Sistla, S. A. *et al.* Long-term warming restructures Arctic tundra without changing net soil carbon storage. *Nature* **497**, 615–619 (2013).
232 Sistla, S. A. & Schimel, J. P. Seasonal patterns of microbial extracellular enzyme activities in an arctic tundra soil: identifying direct and indirect effects of long-term summer warming. *Soil Biol. Biochem.* **66**, 119–129 (2013).

233 Song, H. *et al.* Tropical forest conversion to rubber plantation in southwest China results in lower fungal beta diversity and reduced network complexity. *FEMS Microbiol. Ecol.* **95**, fiz092 (2019).

234 Song, S. *et al.* Responses of wetland soil bacterial community and edaphic factors to two-year experimental warming and *Spartina alterniflora* invasion in Chongming Island. *J. Clean. Prod.* **250**, 119502 (2020).

235 Sonnemann, I. & Wolters, V. The microfood web of grassland soils responds to a moderate increase in atmospheric CO_2. *Glob. Change Biol.* **11**, 1148–1155 (2005).

236 Sui, X. *et al.* Land use change effects on diversity of soil bacterial, Acidobacterial and fungal communities in wetlands of the Sanjiang Plain, northeastern China. *Sci. Rep.* **9**, 1–14 (2019).

237 Suleiman, A. K. A., Manoeli, L., Boldo, J. T., Pereira, M. G. & Roesch, L. F. W. Shifts in soil bacterial community after eight years of land-use change. *Syst. Appl. Microbiol.* **36**, 137–144 (2013).

238 Sun, L. *et al.* Alteration of the soil bacterial community during parent material maturation driven by different fertilization treatments. *Soil Biol. Biochem.* **96**, 207–215 (2016).

239 Sun, R. B., Zhang, X. X., Guo, X. S., Wang, D. Z. & Chu, H. Y. Bacterial diversity in soils subjected to long-term chemical fertilization can be more stably maintained with the addition of livestock manure than wheat straw. *Soil Biol. Biochem.* **88**, 9–18 (2015).

240 Sun, X. *Responses of microbial community taxonomic composition and functional genes to precipitation changes in semi-arid grasslands*. Tsinghua University (2014).

241 Sun, Y. *et al.* Land-use changes alter soil bacterial composition and diversity in tropical forest soil in China. *Sci. Total Environ.* **712**, 136526 (2020).

242 Szoboszlay, M., Dohrmann, A. B., Poeplau, C., Don, A. & Tebbe, C. C. Impact of land-use change and soil organic carbon quality on microbial diversity in soils across Europe. *FEMS Microbiol. Ecol.* **93**, fix146 (2017).

243 Tan, H. *et al.* Long-term phosphorus fertilisation increased the diversity of the total bacterial community and the *phoD* phosphorus mineraliser group in pasture soils. *Biol. Fertil. Soils* **49**, 661–672 (2013).

244 Tang, Y. *et al.* Different strategies for regulating free-living N_2 fixation in nutrient-amended subtropical and temperate forest soils. *Appl. Soil Ecol.* **136**, 21–29 (2019).

245 Tang, Y. *et al.* Impacts of nitrogen and phosphorus additions on the abundance and community structure of ammonia oxidizers and denitrifying bacteria in Chinese fir plantations. *Soil Biol. Biochem.* **103**, 284–293 (2016).

246 Tao, R., Wakelin, S. A., Liang, Y. & Chu, G. Response of ammonia-oxidizing archaea and bacteria in calcareous soil to mineral and organic fertilizer application and their relative contribution to nitrification. *Soil Biol. Biochem.* **114**, 20–30 (2017).

247 Tao, R., Wakelin, S. A., Liang, Y., Hu, B. & Chu, G. Nitrous oxide emission and denitrifier communities in drip-irrigated calcareous soil as affected by chemical and organic fertilizers. *Sci. Total Environ.* **612**, 739–749 (2018).

248 Teng, Z., Cui, J., Wang, J., Fu, X. & Xu, X. Effect of exogenous nitrogen and phosphorus inputs on the microbe-soil interaction in the secondary *Castanopsis sclerophylla* forest in east China. *Iforest* **11**, 794–801 (2018).

249 Thomson, B. C. *et al.* Soil conditions and land use intensification effects on soil microbial communities across a range of European field sites. *Soil Biol. Biochem.* **88**, 403–413 (2015).

250 Tin, H. S. *et al.* Impact of land-use change on vertical soil bacterial communities in Sabah. *Microb. Ecol.* **75**, 459–467 (2018).

251 Treseder, K. K., Marusenko, Y., Romero-Olivares, A. L. & Maltz, M. R. Experimental warming alters potential function of the fungal community in boreal forest. *Glob. Change Biol.* **22**, 3395–3404 (2016).
252 Tripathi, B. M. *et al.* Tropical soil bacterial communities in Malaysia: pH dominates in the equatorial tropics too. *Microb. Ecol.* **64**, 474–484 (2012).
253 Tripathi, B. M. *et al.* Distinctive tropical forest variants have unique soil microbial communities, but not always low microbial diversity. *Front. Microbiol.* **7**, 376 (2016).
254 Tu, J., Qiao, J., Zhu, Z. W., Li, P. & Wu, L. C. Soil bacterial community responses to long-term fertilizer treatments in *Paulownia* plantations in subtropical China. *Appl. Soil Ecol.* **124**, 317–326 (2018).
255 Turlapati, S. A. *et al.* Chronic N-amended soils exhibit an altered bacterial community structure in Harvard Forest, MA, USA. *FEMS Microbiol. Ecol.* **83**, 478–493 (2013).
256 Turner, B. L. *et al.* Seasonal changes and treatment effects on soil inorganic nutrients following a decade of fertilization in a lowland tropical forest. *Soil Sci. Soc. Am. J.* **77**, 1357−1369 (2013).
257 Turner, C. L., Blair, J. M., Schartz, R. J. & Neel, J. C. Soil N and plant responses to fire, topography, and supplemental N in tallgrass prairie. *Ecology* **78**, 1832–1843 (1997).
258 Waghmode, T. R. *et al.* Response of nitrifier and denitrifier abundance and microbial community structure to experimental warming in an agricultural ecosystem. *Front. Microbiol.* **9** (2018).
259 Wan, S., Norby, R. J., Ledford, J. & Weltzin, J. F. Responses of soil respiration to elevated CO_2, air warming, and changing soil water availability in a model old-field grassland. *Glob. Change Biol.* **13**, 2411–2424 (2007).
260 Wang, C. *Shifts of soil microbial communities in Alaskan Tundra in response to long-term warming*. The University of Oklahoma (2016).
261 Wang, C. *et al.* Impact of 25 years of inorganic fertilization on diazotrophic abundance and community structure in an acidic soil in southern China. *Soil Biol. Biochem.* **113**, 240–249 (2017).
262 Wang, C. T. *et al.* The effect of simulated warming on root dynamics and soil microbial community in an alpine meadow of the Qinghai-Tibet Plateau. *Appl. Soil Ecol.* **116**, 30–41 (2017).
263 Wang, F. *et al.* Long-term nitrogen fertilization elevates the activity and abundance of nitrifying and denitrifying microbial communities in an upland soil: implications for nitrogen loss from intensive agricultural systems. *Front. Microbiol.* **9**, 2424 (2018).
264 Wang, H. *et al.* Experimental warming reduced topsoil carbon content and increased soil bacterial diversity in a subtropical planted forest. *Soil Biol. Biochem.* **133**, 155–164 (2019).
265 Wang, H. *et al.* Contrasting responses of heterotrophic and root-dependent respiration to soil warming in a subtropical plantation. *Agric. For. Meteorol.* **247**, 221–228 (2017).
266 Wang, H. *et al.* Nitrogen addition reduces soil bacterial richness, while phosphorus addition alters community composition in an old-growth N-rich tropical forest in southern China. *Soil Biol. Biochem.* **127**, 22–30 (2018).
267 Wang, H. *et al.* Interactive effects of nitrogen fertilizer and altered precipitation on fungal communities in arid grasslands of northern China. *J. Soils Sediments*, 1–13 (2019).
268 Wang, J. *et al.* Impact of inorganic nitrogen additions on microbes in biological soil crusts. *Soil Biol. Biochem.* **88**, 303–313 (2015).

269 Wang, J. *et al.* Effects of nitrogen and water on soil enzyme activity and soil microbial biomass in *Stipa baicalensis* steppe, Inner Mongolia of North China. *Journal of Agricultural Resources and Environment* **31**, 237–245 (2014).
270 Wang, J. *et al.* Mechanisms driving ecosystem carbon sequestration in a Chinese fir plantation: nitrogen versus phosphorus fertilization. *Eur. J. For. Res.* **138**, 863–873 (2019).
271 Wang, P. *et al.* Shifts in microbial communities in soil, rhizosphere and roots of two major crop systems under elevated CO_2 and O_3. *Sci. Rep.* **7**, 15019 (2017).
272 Wang, Q. *et al.* Impact of 36 years of nitrogen fertilization on microbial community composition and soil carbon cycling-related enzyme activities in rhizospheres and bulk soils in northeast China. *Appl. Soil Ecol.* **136**, 148–157 (2019).
273 Wang, Q. *et al.* Effects of nitrogen and phosphorus inputs on soil bacterial abundance, diversity, and community composition in Chinese fir plantations. *Front. Microbiol.* **9**, 1543 (2018).
274 Wang, R. *et al.* Responses of enzymatic activities within soil aggregates to 9-year nitrogen and water addition in a semi-arid grassland. *Soil Biol. Biochem.* **81**, 159–167 (2015).
275 Wang, R. *et al.* Nitrogen application increases soil respiration but decreases temperature sensitivity: Combined effects of crop and soil properties in a semiarid agroecosystem. *Geoderma* **353**, 320–330 (2019).
276 Wang, S. P. *et al.* Effects of warming and grazing on soil N availability, species composition, and ANPP in an alpine meadow. *Ecology* **93**, 2365–2376 (2012).
277 Wang, Y., Ji, H. & Gao, C. Differential responses of soil bacterial taxa to long-term P, N, and organic manure application. *J. Soils Sediments* **16**, 1046–1058 (2016).
278 Wang, Y. *et al.* Different selectivity in fungal communities between manure and mineral fertilizers: a study in an alkaline soil after 30 years fertilization. *Front. Microbiol.* **9**, 2613 (2018).
279 Wang, Z., Liu, Y., Zhao, L., Zhang, W. & Liu, L. Change of soil microbial community under long-term fertilization in a reclaimed sandy agricultural ecosystem. *Peerj* **7**, e6497 (2019).
280 Wang, Z. *et al.* Response of bacterial communities and plant-mediated soil processes to nitrogen deposition and precipitation in a desert steppe. *Plant Soil*, 1–21 (2020).
281 Wu, K., Xu, W. & Yang, W. Effects of precipitation changes on soil bacterial community composition and diversity in the Junggar desert of Xinjiang, China. *Peerj* **8**, e8433 (2020).
282 Wu, S., Huang, B., Gao, J., Wang, S. & Liao, P. The effects of afforestation on soil bacterial communities in temperate grassland are modulated by soil chemical properties. *Peerj* **7**, e6147 (2019).
283 Wu, Y. *et al.* Ecological clusters based on responses of soil microbial phylotypes to precipitation explain ecosystem functions. *Soil Biol. Biochem.* 107717 (2020).
284 Xia, W., Jia, Z., Bowatte, S. & Newton, P. C. Impact of elevated atmospheric CO_2 on soil bacteria community in a grazed pasture after 12-year enrichment. *Geoderma* **285**, 19–26 (2017).
285 Xiang, X., He, D., He, J. S., Myrold, D. D. & Chu, H. Ammonia-oxidizing bacteria rather than archaea respond to short-term urea amendment in an alpine grassland. *Soil Biol. Biochem.* **107**, 218–225 (2017).
286 Xiao, D. *et al.* Arbuscular mycorrhizal fungi abundance was sensitive to nitrogen addition but diversity was sensitive to phosphorus addition in karst ecosystems. *Biol. Fertil. Soils* **55**, 457–469 (2019).

287 Xiao, D. *et al.* Nitrogen fertilizer and *Amorpha fruticosa* leguminous shrub diversely affect the diazotroph communities in an artificial forage grassland. *Sci. Total Environ.* **711**, 134967 (2020).
288 Xie, L. *et al.* Effects of warming and nitrogen addition on the soil bacterial community in a subtropical Chinese fir plantation. *Forests* **10**, 861 (2019).
289 Xiong, J., Peng, F., Sun, H., Xue, X. & Chu, H. Divergent responses of soil fungi functional groups to short-term warming. *Microb. Ecol.* **68**, 708–715 (2014).
290 Xiong, J. *et al.* Characterizing changes in soil bacterial community structure in response to short-term warming. *FEMS Microbiol. Ecol.* **89**, 281–292 (2014).
291 Xu, J. *et al.* Influence of rice cultivars on soil bacterial microbiome under elevated carbon dioxide. *J. Soils Sediments* **19**, 2485–2495 (2019).
292 Xu, M. *et al.* Land use alters arbuscular mycorrhizal fungal communities and their potential role in carbon sequestration on the Tibetan Plateau. *Sci. Rep.* **7**, 3067 (2017).
293 Xu, Y. *et al.* Variations of soil nitrogen-fixing microorganism communities and nitrogen fractions in a *Robinia pseudoacacia* chronosequence on the Loess Plateau of China. *Catena* **174**, 316–323 (2019).
294 Yang, F., Niu, K., Collins, C. G., Yan, X. & Hu, S. Grazing practices affect the soil microbial community composition in a Tibetan alpine meadow. *Land Degrad. Dev.* **30**, 49–59 (2019).
295 Yang, S. *et al.* Long-termelevated CO_2 shifts composition of soil microbial communities in a Californian annual grassland, reducing growth and N utilization potentials. *Sci. Total Environ.* **652**, 1474–1481 (2019).
296 Yang, X. *et al.* Long-term nitrogen fertilization indirectly affects soil fungi community structure by changing soil and pruned litter in a subtropical tea (*Camellia sinensis L.*) plantation in China. *Plant Soil* **444**, 409–426 (2019).
297 Yang, Y. *et al.* Differentiated responses of *nirS*- and *nirK*-type denitrifiers to 30 years of combined inorganic and organic fertilization in a paddy soil. *Arch. Agron. Soil Sci.* (2020).
298 Yang, Y. *et al.* Ammonia-oxidizing archaea and bacteria responding differently to fertilizer type and irrigation frequency as revealed by Illumina Miseq sequencing. *J. Soils Sediments* **18**, 1029–1040 (2018).
299 Yang, Y., Wang, P. & Zeng, Z. Dynamics of bacterial communities in a 30-year fertilized paddy field under different organic-inorganic fertilization strategies. *Agronomy* **9**, 14 (2019).
300 Yang, Y., Wang, Z. & Zeng, Z. Effects of long-term different fertilization and irrigation managements on soil bacterial abundance, diversity and composition. *Scientia Agricultura Sinica* **51**, 290–301 (2018).
301 Yao, L. H. *et al.* Effects of fertilizations on soil bacteria and fungi communities in a degraded arid steppe revealed by high through-put sequencing. *Peerj* **6**, e4623 (2018).
302 Ye, C., Chen, C., Du, M., Liu, W. & Zhang, Q. Revegetation affects soil denitrifying communities in a riparian ecotone. *Ecol. Eng.* **103**, 256–263 (2017).
303 Yergeau, E. *et al.* Shifts in soil microorganisms in response to warming are consistent across a range of Antarctic environments. *ISME J.* **6**, 692–702 (2012).
304 Yu, C., Han, F. & Fu, G. Effects of 7 years experimental warming on soil bacterial and fungal community structure in the Northern Tibet alpine meadow at three elevations. *Sci. Total Environ.* **655**, 814–822 (2019).
305 Yu, C. Q., Shen, Z. X., Zhang, X. Z., Sun, W. & Fu, G. Response of soil C and N, dissolved organic C and N, and inorganic N to short-term experimental warming in an alpine meadow on the Tibetan Plateau. *Sci. World J.* **2014** (2014).

306 Yu, H. *et al.* Responses of soil biological traits and bacterial communities to nitrogen fertilization mediate maize yields across three soil types. *Soil Till. Res.* **185**, 61–69 (2019).
307 Yu, Y. *et al.* Responses of paddy soil bacterial community assembly to different long-term fertilizations in southeast China. *Sci. Total Environ.* **656**, 625–633 (2019).
308 Yu, Y. J. *et al.* Divergent responses of the diazotrophic microbiome to elevated CO_2 in two rice cultivars. *Front. Microbiol.* **9**, 1139 (2018).
309 Yu, Z. *et al.* Effectiveness of elevated CO_2 mediating bacterial communities in the soybean rhizosphere depends on genotypes. *Agric. Ecosyst. Environ.* **231**, 229–232 (2016).
310 Yu, Z. *et al.* Impact of land use, fertilization and seasonal variation on the abundance and diversity of *nirS*-type denitrifying bacterial communities in a Mollisol in Northeast China. *Eur. J. Soil Biol.* **85**, 4–11 (2018).
311 Yu, Z. *et al.* Responses of ammonia-oxidizing bacterial communities to land-use and seasonal changes in Mollisols of Northeast China. *Eur. J. Soil Biol.* **74**, 121–127 (2016).
312 Yuan, J., Yuan, Y., Zhu, Y. & Cao, L. Effects of different fertilizers on methane emissions and methanogenic community structures in paddy rhizosphere soil. *Sci. Total Environ.* **627**, 770–781 (2018).
313 Yuan, X. *et al.* Plant community and soil chemistry responses to long-term nitrogen inputs drive changes in alpine bacterial communities. *Ecology* **97**, 1543–1554 (2016).
314 Zeglin, L. H., Taylor, A. E., Myrold, D. D. & Bottomley, P. J. Bacterial and archaeal *amoA* gene distribution covaries with soil nitrification properties across a range of land uses. *Environ. Microbiol. Rep.* **3**, 717–726 (2011).
315 Zeng, J. *et al.* Nitrogen fertilization directly affects soil bacterial diversity and indirectly affects bacterial community composition. *Soil Biol. Biochem.* **92**, 41–49 (2016).
316 Zhang, G. *et al.* Ammonia-oxidizing bacteria and archaea: response to simulated climate warming and nitrogen supplementation. *Soil Sci. Soc. Am. J.* **83**, 1683–1695 (2019).
317 Zhang, H. *et al.* Elevated precipitation modifies the relationship between plant diversity and soil bacterial diversity under nitrogen deposition in *Stipa baicalensis* steppe. *Appl. Soil Ecol.* **119**, 345–353 (2017).
318 Zhang, H. *et al.* Conversion from natural wetlands to forestland and farmland alters the composition of soil fungal communities in Sanjiang Plain, Northeast China. *Biotechnol. Biotechnol. Equip.* **32**, 951–960 (2018).
319 Zhang, K. *et al.* Effects of short-term warming and altered precipitation on soil microbial communities in alpine grassland of the Tibetan plateau. *Front. Microbiol.* **7**, 1032 (2016).
320 Zhang, W. *et al.* Soil microbial responses to experimental warming and clipping in a tallgrass prairie. *Glob. Change Biol.* **11**, 266–277 (2005).
321 Zhang, X., Johnston, E. R., Li, L., Konstantinidis, K. T. & Han, X. Experimental warming reveals positive feedbacks to climate change in the Eurasian Steppe. *ISME J.* **11**, 885–895 (2017).
322 Zhang, X., Johnston, E. R., Liu, W., Li, L. & Han, X. Environmental changes affect the assembly of soil bacterial community primarily by mediating stochastic processes. *Glob. Change Biol.* **22**, 198–207 (2016).
323 Zhang, X. *et al.* Distinct drivers of core and accessory components of soil microbial community functional diversity under environmental changes. *MSystems* **4**, e00374–00319 (2019).

324 Zhang, X., Liu, W., Bai, Y., Zhang, G. & Han, X. Nitrogen deposition mediates the effects and importance of chance in changing biodiversity. *Mol. Ecol.* **20**, 429–438 (2011).
325 Zhang, Y. *et al.* Soil bacterial and fungal diversity differently correlated with soil biochemistry in alpine grassland ecosystems in response to environmental changes. *Sci. Rep.* **7**, 43077 (2017).
326 Zhang, Y. *et al.* Climate change and human activities altered the diversity and composition of soil microbial community in alpine grasslands of the Qinghai-Tibetan Plateau. *Sci. Total Environ.* **562**, 353–363 (2016).
327 Zhang, Y. *et al.* Fertilization shapes bacterial community structure by alteration of soil pH. *Front. Microbiol.* **8**, 1325 (2017).
328 Zhao, A. *et al.* Influences of canopy nitrogen and water addition on AM fungal biodiversity and community composition in a mixed deciduous forest of China. *Front. Plant Sci.* **9**, 1842 (2018).
329 Zhao, C. C. *et al.* Soil microbial community composition and respiration along an experimental precipitation gradient in a semiarid steppe. *Sci. Rep.* **6**, 24317 (2016).
330 Zheng, H. *et al.* Short-term warming shifts microbial nutrient limitation without changing the bacterial community structure in an alpine timberline of the eastern Tibetan Plateau. *Geoderma* **360**, 113985 (2020).
331 Zheng, Y. *et al.* Differential responses of arbuscular mycorrhizal fungi to nitrogen addition in a near pristine Tibetan alpine meadow. *FEMS Microbiol. Ecol.* **89**, 594–605 (2014).
332 Zheng, Y. *et al.* Methanotrophic community structure and activity under warming and grazing of alpine meadow on the Tibetan Plateau. *Appl. Microbiol. Biotechnol.* **93**, 2193–2203 (2012).
333 Zhong, Y., Yan, W. & Shangguan, Z. Impact of long-term N additions upon coupling between soil microbial community structure and activity, and nutrient-use efficiencies. *Soil Biol. Biochem.* **91**, 151–159 (2015).
334 Zhou, H. *et al.* Changes in the soil microbial communities of alpine steppe at Qinghai-Tibetan Plateau under different degradation levels. *Sci. Total Environ.* **651**, 2281–2291 (2019).
335 Zhou, J. *et al.* Influence of 34-years of fertilization on bacterial communities in an intensively cultivated black soil in northeast China. *Soil Biol. Biochem.* **90**, 42–51 (2015).
336 Zhou, J. *et al.* Thirty four years of nitrogen fertilization decreases fungal diversity and alters fungal community composition in black soil in northeast China. *Soil Biol. Biochem.* **95**, 135–143 (2016).
337 Zhou, J. *et al.* Microbial mediation of carbon-cycle feedbacks to climate warming. *Nat. Clim. Change* **2**, 106–110 (2012).
338 Zhou, X. *et al.* Warming and increased precipitation have differential effects on soil extracellular enzyme activities in a temperate grassland. *Sci. Total Environ.* **444**, 552–558 (2013).
339 Zhou, X. *et al.* Effects of 44 years of chronic nitrogen fertilization on the soil nitrifying community of permanent grassland. *Soil Biol. Biochem.* **91**, 76–83 (2015).
340 Zhu, C. *et al.* N-fertilizer-driven association between the arbuscular mycorrhizal fungal community and diazotrophic community impacts wheat yield. *Agric. Ecosyst. Environ.* **254**, 191–201 (2018).
341 Zi, H. B. *et al.* Responses of soil bacterial community and enzyme activity to experimental warming of an alpine meadow. *Eur. J. Soil Sci.* **69**, 429–438 (2018).
