# Supplementary material for: Meta-analysis of the impacts of global change factors on soil microbial diversity and functionality
Source: Nat Commun. 2020 Jun 17;11:3072. doi: 10.1038/s41467-020-16881-7 (PMC7300008; doi:10.1038/s41467-020-16881-7)
Supplement: Supplementary file 3 — Reporting Summary [file 41467_2020_16881_MOESM3_ESM.pdf]

nature research

Corresponding author(s): Zhenghu Zhou, Chuankuan Wang

Last updated by author(s): May 21, 2020

Reporting Summary

Nature Research wishes to improve the reproducibility of the work that we publish. This form provides structure for consistency and transparency in reporting. For further information on Nature Research policies, see Authors & Referees and the Editorial Policy Checklist .

Statistics

For all statistical analyses, confirm that the following items are present in the figure legend, table legend, main text, or Methods section.

n/a	Confirmed	
☐	☒	The exact sample size (*n*) for each experimental group/condition, given as a discrete number and unit of measurement
☐	☒	A statement on whether measurements were taken from distinct samples or whether the same sample was measured repeatedly
☐	☒	The statistical test(s) used AND whether they are one- or two-sided<br>*Only common tests should be described solely by name; describe more complex techniques in the Methods section.*
☐	☒	A description of all covariates tested
☐	☒	A description of any assumptions or corrections, such as tests of normality and adjustment for multiple comparisons
☐	☒	A full description of the statistical parameters including central tendency (e.g. means) or other basic estimates (e.g. regression coefficient) AND variation (e.g. standard deviation) or associated estimates of uncertainty (e.g. confidence intervals)
☐	☒	For null hypothesis testing, the test statistic (e.g. *F*, *t*, *r*) with confidence intervals, effect sizes, degrees of freedom and *P* value noted<br>*Give P values as exact values whenever suitable.*
☒	☐	For Bayesian analysis, information on the choice of priors and Markov chain Monte Carlo settings
☒	☐	For hierarchical and complex designs, identification of the appropriate level for tests and full reporting of outcomes
☐	☒	Estimates of effect sizes (e.g. Cohen's *d*, Pearson's *r*), indicating how they were calculated

Our web collection on statistics for biologists contains articles on many of the points above.

Software and code

Policy information about availability of computer code

Data collection	An extensive literature survey was conducted through the Google Scholar databases. The authors declare that the data supporting the findings of this study are available in Supplementary Dataset 1 and 2.
Data analysis	All analyses were conducted in the R-3.6.2. The authors declare that the R codes used to generate the results and figures reported in this study are available in Supplementary Code 1.

For manuscripts utilizing custom algorithms or software that are central to the research but not yet described in published literature, software must be made available to editors/reviewers. We strongly encourage code deposition in a community repository (e.g. GitHub). See the Nature Research guidelines for submitting code & software for further information.

Data

Policy information about availability of data

All manuscripts must include a data availability statement. This statement should provide the following information, where applicable:

- Accession codes, unique identifiers, or web links for publicly available datasets
- A list of figures that have associated raw data
- A description of any restrictions on data availability

An extensive literature survey was conducted through the Google Scholar Database (https://scholar.google.com/). The authors declare that the data supporting the findings of this study are available in Supplementary Dataset 1 and 2.

Field-specific reporting

Please select the one below that is the best fit for your research. If you are not sure, read the appropriate sections before making your selection.

Ecological, evolutionary & environmental sciences study design

All studies must disclose on these points even when the disclosure is negative.

Study description	A meta-analysis was conducted to examine effects of global changes (climate warming, carbon dioxide enrichment, altered precipitation, atmospheric nitrogen deposition, nutrient fertilization, land use change, and their combinations) on soil microbial diversity and functionality.
Research sample	Total of 1235 GCF experimental observations that measured microbial alpha diversity (richness and Shannon index), beta diversity, and community structure with high-throughput sequencing techniques, and corresponding biomass and ecosystem functionalities from 341 publications were collected by searching Google Scholar Database (https://scholar.google.com/).
Sampling strategy	Literature search and scanning of papers for adherence to a priori inclusion criteria.
Data collection	Literature search on Google Scholar databases by ZZ.
Timing and spatial scale	All papers published by February 2020 included (no start date).
Data exclusions	No data that met the a priori inclusion criteria were excluded.
Reproducibility	Data were extracted and analyzed following the protocols described in the methods and R code.
Randomization	Randomization is not applicable to a meta-analysis.
Blinding	Blinding is not applicable to a meta-analysis.

Did the study involve field work? ☐ Yes ☒ No

Reporting for specific materials, systems and methods

We require information from authors about some types of materials, experimental systems and methods used in many studies. Here, indicate whether each material, system or method listed is relevant to your study. If you are not sure if a list item applies to your research, read the appropriate section before selecting a response.

Materials & experimental systems

n/a	Involved in the study
☒	☐ Antibodies
☒	☐ Eukaryotic cell lines
☒	☐ Palaeontology
☒	☐ Animals and other organisms
☒	☐ Human research participants
☒	☐ Clinical data

Methods

n/a	Involved in the study
☒	☐ ChIP-seq
☒	☐ Flow cytometry
☒	☐ MRI-based neuroimaging
