# Supplementary material for: Meta-analysis of the impacts of global change factors on soil microbial diversity and functionality
Source: Nat Commun. 2020 Jun 17;11:3072. doi: 10.1038/s41467-020-16881-7 (PMC7300008; doi:10.1038/s41467-020-16881-7)
Supplement: Supplementary file 4 — Description of Additional Supplementary Files [file 41467_2020_16881_MOESM4_ESM.pdf]

Description of Additional Supplementary Files

File Name: Supplementary Data 1
Description: The dataset supporting the findings of this study for site, microbial alpha diversity and function.

File Name: Supplementary Data 2
Description: The dataset supporting the findings of this study for microbial beta diversity and community composition.

File Name: Supplementary Data 3
Description: A table summarizing basic characteristics of the dataset of this study.

File Name: Supplementary Software 1
Description: The R codes used to generate the results and figures reported in the paper.
